# Synthesis and Decontamination Effect on Chemical and Biological Agents of Benzoxonium-Like Salts

**DOI:** 10.3390/toxics9090222

**Published:** 2021-09-15

**Authors:** Aneta Markova, Michaela Hympanova, Marek Matula, Lukas Prchal, Radek Sleha, Marketa Benkova, Lenka Pulkrabkova, Ondrej Soukup, Zuzana Krocova, Daniel Jun, Jan Marek

**Affiliations:** 1 Biomedical Research Centre, University Hospital Hradec Kralove, Sokolska 581, 500 05 Hradec Kralove, Czech Republic; aneta.markova@fnhk.cz (A.M.); michaela.hympanova@fnhk.cz (M.H.); lukas.prchal@fnhk.cz (L.P.); Marketa.Benkova@fnhk.cz (M.B.); lenka.pulkrabkova@fnhk.cz (L.P.); ondrej.soukup@fnhk.cz (O.S.); 2 Department of Toxicology and Military Pharmacy, Faculty of Military Health Sciences, University of Defence in Brno, Trebesska 1575, 500 05 Hradec Kralove, Czech Republic; marek.matula@unob.cz (M.M.); daniel.jun@unob.cz (D.J.); 3 Department of Epidemiology, Faculty of Military Health Sciences, University of Defence in Brno, Trebesska 1575, 500 05 Hradec Kralove, Czech Republic; radek.sleha@unob.cz; 4 Department of Molecular Pathology and Biology, Faculty of Military Health Sciences, University of Defence in Brno, Trebesska 1575, 500 05 Hradec Kralove, Czech Republic; zuzana.krocova@unob.cz

**Keywords:** quaternary ammonium salts, benzoxonium, decontamination, organophosphates, disinfection, micellar catalysis

## Abstract

Benzoxonium chloride belongs to the group of quaternary ammonium salts, which have been widely used for decades as disinfectants because of their high efficacy, low toxicity, and thermal stability. In this study, we have prepared the C_10_-C_18_ set of benzoxonium-like salts to evaluate the effect of their chemical and biological decontamination capabilities. In particular, biocidal activity against a panel of bacterial strains including *Staphylococcus aureus* in biofilm form was screened. In addition, the most promising compounds were successfully tested against *Francisella tularensis* as a representative of potential biological warfare agents. From a point of view of chemical warfare protection, the efficiency of BOC-like compounds to degrade the organophosphate simulant fenitrothion was examined. Notwithstanding that no single compound with universal effectiveness was identified, a mixture of only two compounds from this group would be able to satisfactorily cover the proposed decontamination spectrum. In addition, the compounds were evaluated for their cytotoxicity as a basic safety parameter for potential use in practice. In summary, the dual effect on chemical and biological agents of benzoxonium-like salts offer attractive potential as active components of decontamination mixtures in the case of a terrorist threat or chemical or biological accidents.

## 1. Introduction

Benzoxonium chloride (N-benzyl-N,N-bis(2-hydroxyethyl)dodecan-1-aminium chloride, BOC), known for over 30 years [1], manifests strong antimicrobial activity against certain bacterial strains, especially non-spore forming, yeasts and protozoa. In practice, BOC is widely used against buccopharyngeal pathogens as a mucous membrane disinfectant. It is commercially available in the form of gargle solutions, lozenges, and oral sprays, often in combination with lidocaine [2,3]. Likewise in dentistry it is used against dental plaque and dental caries [4,5]. Further applications include disinfection of medical instruments in hospitals, and of skin burns, seborrheic dermatitis, and cutaneous leishmaniosis [4,6,7], as a veterinary fungicide and bactericide in the treatment of animal skin, in the food and beverage industry, and in the textile industry as a bactericide in textile fibers [1,8,9]. Of note, BOC is one of the few quaternary ammonium salts (together with cetrimide and benzalkonium chloride (BAC)) highly efficient in preservation of topical eye medications [10,11].

BOC belongs in the wide group of quaternary ammonium salts (QACs)—substances with surface-active properties. They are still considered an important component of hygienic and anti-epidemic measures against the spread of nosocomial diseases, due to their high efficacy, low price, and only modest toxicity to eukaryotic cells [12,13].

QACs are predominantly supposed to have relatively unspecific mechanisms of antimicrobial action. Owing to the positively charged nitrogen moiety, QACs are able to interact with the negatively charged outermost surface of cell walls and membranes and penetrate into the phospholipid bilayer with their long alkyl chain, thus causing leakages, ruptures, and disintegration of the cell [14]. QACs exhibit higher activity against Gram-positive (G+) bacteria as these possess a single phospholipid cellular membrane and a thicker cell wall composed of peptidoglycan, whereas Gram-negative (G-) bacteria are enveloped by two cellular membranes and a thinner layer of peptidoglycan. The outer membrane causes decreased antimicrobial activity in the case of Gram-negative bacteria [15].

However, recent studies show that some QACs can be also used as micellar catalysts because of their ability to accelerate the decomposition of neurotoxic organophosphate esters [16,17,18,19,20]. Such esters (e.g., fenitrothion, chlorpyrifos, paraoxon) are widely used as pesticides in agriculture. Similarly, highly toxic organophosphates, so-called nerve agents (tabun, soman and sarin and VX), have been reported be also susceptible to decomposition of their organophosphorus ester moieties by quaternary ammonium salt-type cationic surfactants.

The above-mentioned properties of QACs render benzoxonium salts as suitable active ingredients of a decontamination mixture against chemical and biological agents. Despite the fact that BOC is a well-known compound, there is a lack of comprehensive information describing its potential decontamination properties, and the preparation and evaluation of the whole series [21].

Therefore, within this study, we have designed and synthesized a novel series of BOC-like compounds differing in the long alkyl chain (C_10_-C_18_) with an expected dual effect on biological and chemical agents including agents with warfare potential. The decontamination properties screening assessed the antimicrobial effect against eight floating-form strains and a representative biofilm-forming strain of *S. aureus* [22]. The cells in biofilms possess different characteristics than single cells and due to their specific structure present decreased susceptibility [23,24]. We further focused on potentially misused warfare agents. Antimicrobial activity was determined against the Gram-negative *Francisella tularensis*, with potential to be misused as a biological weapon; also determined was the efficiency of BOC-like compounds to decompose fenitrothion as an organophosphate simulant. Finally, the cytotoxicity on a mammalian cell line was evaluated as one of the key safety parameters for new substances. Despite the above-mentioned properties, the substances can have practical use in the military (the main active ingredient of a universal decontamination mixture for chemical and biological warfare agents in form of solution or foam). Further, in the civil sector use as disinfectant in healthcare (viruses, bacteria, fungi) or as active ingredients for decontamination of organophosphorus pesticides (from water, soil, etc.).

## 2. Materials and Methods

### 2.1. Synthesis and Analysis

Analytical grade reagents were purchased from Sigma-Aldrich, Fluka, and Merck (Darmstadt, Germany). The solvents were purchased from Penta Chemicals (Prague, Czech Republic). Reactions were monitored by thin-layer chromatography (TLC) using pre-coated silica gel 60 F254 TLC alumina sheet. Column chromatography was performed with silica gel 0.063e0.200 mm. Melting points (m. p.) were determined on a micro heating stage PHMK 05 (VEB Kombinat Nagema, Radebeul, Germany) and are uncorrected. All compounds were fully characterized by NMR spectra and HRMS. NMR spectra were recorded on a Varian VNMR S500 (operating at 500 MHz for ^1^H and 125 MHz for ^13^C; Varian Co., Palo Alto, CA, USA). The chemical shifts (d) are given in ppm, relative to tetramethylsilane (TMS) as internal standard. Coupling constants (*J*) are reported in Hz. Splitting patterns are designated as s, singlet; d, doublet; t, triplet; and m, multiplet. Mass spectra were recorded using a combination of liquid chromatography and mass spectrometry: high-resolution mass spectra (HRMS) and sample purities were obtained by high-performance liquid chromatography (HPLC) with UV and mass spectrometric (MS) detection gradient method. The system used in this study was a Dionex Ultimate 3000 UHPLC: RS Pump, RS Column Compartment, RS Autosampler, Diode Array Detector, controlled by Chromeleon (version 7.2.9 build 11323) software (Thermo Fisher Scientific, Germering, Germany) with Q Exactive Plus Orbitrap mass spectrometer with Thermo Xcalibur (version 3.1.66.10.) software (Thermo Fisher Scientific, Bremen, Germany). Detection was performed by mass spectrometer in positive mode. Settings of the heated electrospray source were: Spray voltage 3.5 kV, Capillary temperature: 262 °C, Sheath gas: 55 arbitrary units, Auxiliary gas: 15 arbitrary units, Spare gas: 3 arbitrary units, Probe heater temperature: 250 °C, Max spray current: 100 mA, S-lens RF Level: 50. C18 column (Kinetex EVO C18, 3 × 150 mm, 2.6 µm, Phenomenex, Torrance, CA, USA) was used in this study. Mobile phase A was ultrapure water of ASTM I type (resistivity 18.2 MΩ.cm at 25 °C) prepared by Barnstead Smart2Pure 3 UV/UF apparatus (Thermo Fisher Scientific, Bremen, Germany) with 0.1% (*v*/*v*) formic acid (HPLC-MS grade, Sigma Aldrich, Steinheim, Germany); mobile phase B was acetonitrile (MS grade, Honeywell-Sigma Aldrich) with 0.1% (*v*/*v*) of formic acid. The flow was constant at 0.4 mL/min. The method started with 1 min of isocratic flow of 5% B, and then the gradient of B rose to 100% B in 3 min and remained constant at 100% B for 1 min. The composition then went back to 5% B and equilibrated for 5 min. Total runtime of the method was 10 min. The column was heated to 27 °C. Samples were dissolved in methanol (LC-MS grade, Fluka-Sigma Aldrich) at a concentration 1 mg/mL and sample injection was 1 µL. Purity was determined from UV spectra measured at wavelength 254 nm. HRMS was determined by total ion current spectra from the mass spectrometer collected at resolution of 140,000 in a range 105–1000 *m/z*. Clog *P* was calculated with the MarvinSketch (version 14.9.8.0) software.

#### 2.1.1. General Procedure for Synthesis of N,N-bis(2-hydroxyethyl)-N-alkylamines (**3a-e**)

Diethanolamine (**1**) (22.5 mmol), anhydrous potassium carbonate (30 mmol), and potassium iodide (3 mmol) were dispersed in 40 mL of acetonitrile. Subsequently, the appropriate 1-bromoalkane **2a-e** (15 mmol) was added. The reaction mixture was refluxed under nitrogen atmosphere for 12 h. After filtration the acetonitrile was evaporated under reduced pressure. The residue was dissolved in water (30 mL) and extracted twice with dichloromethane. The organic layer was dried over anhydrous sodium sulfate, filtered, and evaporated to obtain a yellow oily product in 65–96% yields. The intermediates **3a-e** were of satisfactory purity for the next reaction step (>95%).

#### 2.1.2. General Procedures for Synthesis of N-benzyl-N,N-bis(2-hydroxyethyl)alkane-1-aminium Chloride (**5a-e**)

A mixture of the appropriate intermediate *N*,*N*-bis(2-hydroxyethyl)-*N*-alkylamine **3a-e** (14 mmol) and benzyl chloride (**4**, 28 mmol) was heated in a 100 mL flask at 100 °C for 2 h as a solvent-free reaction. Subsequently, the reaction was allowed to stand for 5 days at room temperature. Crude products were purified by crystallization in a medley of 1:10 methanol/ethyl acetate and subsequently in 1:10 methanol/diethyl ether to obtain white solid in 42–79% yields.

#### 2.1.3. NMR and HRMS Analysis

*N,N-Bis(2-hydroxyethyl)-N-decylamine* (**3a**). Yellow oil, (2.40 g, 65%); ^1^H-NMR (chloroform-*d*) δ 3.63 (t, *J* = 5.4 Hz, 4H), 2.68 (t, *J* = 5.4 Hz, 4H), 2.57–2.50 (m, 2H), 1.51–1.44 (m, 2H), 1.35–1.21 (m, 14H), 0.89 (t, *J* = 6.8 Hz, 3H); ^13^C-NMR (chloroform-*d*) δ 59.67, 56.05, 54.79, 31.88, 29.61, 29.55, 29.30, 27.36, 27.17, 22.67, 14.10; HRMS: *m/z* 246.2424 [M + H]^+^ (calculated *m/z* 246.2428 for [C_14_H_32_NO_2_]).

*N,N-Bis(2-hydroxyethyl)-N-dodecylamine* (**3b**). Yellow oil, (3.75 g, 91%); ^1^H-NMR (chloroform-*d*) δ 3.63 (t, *J* = 5.4 Hz, 4H), 2.68 (t, *J* = 5.4 Hz, 4H), 2.58–2.51 (m, 2H), 1.52–1.44 (m, 2H), 1.32–1.22 (m, 18H), 0.89 (t, *J* = 7.1, 1.7 Hz, 3H); ^13^C-NMR (chloroform-*d*) δ 59.46, 56.08, 54.80, 31.87, 29.62, 29.59, 29.52, 29.30, 27.33, 26.80, 22.64, 14.07; HRMS: *m/z* 274.2736 [M + H]^+^ (calculated *m/z* 274.2741 for [C_16_H_36_NO_2_]).

*N,N-Bis(2-hydroxyethyl)-N-tetradecylamine* (**3c**). Yellow oil, (3.07 g, 67%); ^1^H-NMR (chloroform-*d*) δ 3.62 (t, *J* = 5.4 Hz, 4H), 2.66 (t, *J* = 5.4 Hz, 4H), 2.55–2.49 (m, 2H), 1.49–1.43 (m, 2H), 1.32–1.22 (m, 22H), 0.88 (t, *J* = 6.9 Hz, 3H); ^13^C-NMR (chloroform-*d*) δ 59.65, 56.05, 54.79, 31.90, 29.67, 29.65, 29.63, 29.62, 29.57, 29.56, 29.34, 27.37, 27.08, 22.67, 14.09; HRMS: *m/z* 302.3054 [M + H]^+^ (calculated *m/z* 302.3054 for [C_18_H_40_NO_2_]).

*N,N-Bis(2-hydroxyethyl)-N-hexadecylamine* (**3d**). Yellow oil, (3.82 g, 77%); ^1^H-NMR (chloroform-*d*) δ 3.62 (t, *J* = 5.4 Hz, 4H), 2.66 (t, *J* = 5.4 Hz, 4H), 2.56–2.49 (m, 2H), 1.51–1.43 (m, 2H), 1.29–1.25 (m, 26H), 0.89 (t, *J* = 6.9 Hz, 3H); ^13^C-NMR (chloroform-*d*) δ 59.65, 56.05, 54.79, 31.91, 29.68, 29.66, 29.64, 29.62, 29.56, 29.34, 27.37, 27.09, 22.67, 14.09; HRMS: *m/z* 330.3361 [M + H]^+^ (calculated *m/z* 330.3367 for [C_20_H_44_NO_2_]).

*N,N-Bis(2-hydroxyethyl)-N-octadecylamine* (**3e**). Yellow solid, m. p. 48.1–49.7 °C (5.17 g, 96%); ^1^H-NMR (chloroform-*d*) δ 3.62 (t, *J* = 5.4 Hz, 4H), 2.66 (t, *J* = 5.4 Hz, 4H), 2.55–2.49 (m, 2H), 1.49–1.43 (m, 2H), 1.29–1.24 (m, 30H), 0.88 (t, *J* = 6.9 Hz, 3H); ^13^C-NMR (chloroform-*d*) δ 61.10, 59.62, 56.04, 54.78, 50.94, 31.90, 29.67, 29.64, 29.61, 29.56, 29.33, 27.37, 27.00, 22.66, 14.09; HRMS: *m/z* 358.3675 [M + H]^+^ (calculated *m/z* 358.3680 for [C_22_H_48_NO_2_]).

*N-benzyl-N,N-bis(2-hydroxyethyl)decan-1-aminium chloride* (**5a**). White solid, m. p. 114.0–114.6 °C (4.09 g, 78%); ^1^H-NMR (chloroform-*d*) δ 7.63–7.57 (m, 2H), 7.47–7.39 (m, 3H), 5.73 (t, *J* = 5.4 Hz, 2H), 4.86 (s, 2H), 4.29–4.10 (m, 4H), 3.71–3.52 (m, 4H), 3.39–3.32 (m, 2H), 1.89–1.80 (m, 2H), 1.38–1.14 (m, 14H), 0.87 (t, *J* = 6.9 Hz, 3H); ^13^C-NMR (chloroform-*d*) δ 133.18, 130.61, 129.25, 127.27, 64.39, 60.20, 59.60, 55.83, 31.78, 29.37, 29.36, 29.18, 29.13, 26.22, 22.69, 22.59, 14.05; HRMS: *m/z* 336.2888 [M]^+^ (calculated *m/z* 336.2897 for [C_21_H_38_NO_2_]^+^).

*N-benzyl-N,N-bis(2-hydroxyethyl)dodecan-1-aminium chloride* (**5b**). White solid, m. p. 118.7–119.0 °C (4.68 g, 41%); ^1^H-NMR (chloroform-*d*) δ 7.62–7.57 (m, 2H), 7.47–7.39 (m, 3H), 5.30 (t, *J* = 5.4 Hz, 2H), 4.86 (s, 2H), 4.27–4.14 (m, 4H), 3.71–3.53 (m, 4H), 3.38–3.31 (m, 2H), 1.89–1.80 (m, 2H), 1.36–1.19 (m, 18H), 0.88 (t, *J* = 6.9 Hz, 3H); ^13^C-NMR (chloroform-*d*) δ 133.17, 130.61, 129.26, 127.27, 64.39, 60.21, 59.61, 55.83, 31.84, 29.55, 29.42, 29.39, 29.27, 29.15, 26.23, 22.69, 22.62, 14.06; HRMS: *m/z* 364.3207 [M]^+^ (calculated *m/z* 364.3210 for [C_23_H_42_NO_2_]^+^).

*N-benzyl-N,N-bis(2-hydroxyethyl)tetradecan-1-aminium chloride* (**5c**). White solid, m. p. 120.6–120.9 °C (3.92 g, 65%); ^1^H-NMR (methanol-*d*_4_) δ 7.68–7.62 (m, 2H), 7.60–7.49 (m, 3H), 4.79 (s, 2H), 4.09 (t, *J* = 5.1 Hz, 4H), 3.51 (t, *J* = 7.4, 5.0 Hz, 4H), 3.38–3.31 (m, 2H), 1.98–1.88 (m, 2H), 1.45–1.28 (m, 22H), 0.90 (t, *J* = 6.9 Hz, 3H); ^13^C-NMR (methanol-*d*_4_) δ 134.34, 131.79, 130.36, 129.11, 65.40, 61.34, 61.08, 56.71, 33.06, 30.79, 30.77, 30.75, 30.71, 30.61, 30.53, 30.46, 30.18, 27.34, 23.72, 23.49, 14.44; HRMS: *m/z* 392.3515 [M]^+^ (calculated *m/z* 392.3523 for [C_25_H_46_NO_2_]^+^).

*N-benzyl-N,N-bis(2-hydroxyethyl)hexadecan-1-aminium chloride* (**5d**). White solid, m. p. 121.6–121.9 °C (4.39 g, 68%); ^1^H-NMR (chloroform-*d*) δ 7.63–7.57 (m, 2H), 7.48–7.40 (m, 3H), 5.73 (t, *J* = 5.4 Hz, 2H), 4.86 (s, 2H), 4.28–4.14 (m, 4H), 3.69–3.55 (m, 4H), 3.39–3.32 (m, 2H), 1.90–1.80 (m, 2H), 1.32–1.23 (m, 26H), 0.87 (t, *J* = 6.9 Hz, 3H); ^13^C-NMR (chloroform-*d*) δ 133.22, 130.67, 129.31, 127.31, 64.43, 60.25, 59.65, 55.89, 31.92, 29.70, 29.66, 29.61, 29.48, 29.45, 29.36, 29.21, 26.28, 22.75, 22.68, 14.11; HRMS: *m/z* 420.3828 [M]^+^ (calculated *m/z* 420.3836 for [C_27_H_50_NO_2_]^+^).

*N-benzyl-N,N-bis(2-hydroxyethyl)octadecan-1-aminium chloride* (**5e**). White solid, m. p. 124.2–124.7 °C (3.59 g, 52%); ^1^H-NMR (chloroform-*d*) δ 7.62–7.57 (m, 2H), 7.49–7.39 (m, 3H), 5.72 (t, *J* = 5.4 Hz, 2H), 4.87 (s, 2H), 4.22 (t, *J* = 10.8, 6.3, 5.6 Hz, 4H), 3.62 (t, *J* = 39.6, 14.4, 6.5, 3.3 Hz, 4H), 3.38–3.31 (m, 2H), 1.90–1.81 (m, 2H), 1.38–1.15 (m, 30H), 0.88 (t, *J* = 6.8 Hz, 3H); ^13^C-NMR (chloroform-*d*) δ 133.19, 130.63, 129.28, 127.28, 64.40, 60.22, 59.61, 55.87, 31.88, 29.68, 29.64, 29.62, 29.59, 29.46, 29.42, 29.32, 29.18, 26.24, 22.71, 22.65, 14.08; HRMS: *m/z* 448.4153 [M]^+^ (calculated *m/z* 448.4149 for [C_29_H_54_NO_2_]^+^).

All spectra are available in the Supplementary Materials (Figures S1–S15).

### 2.2. Conductivity

The conductivity of surfactant solutions was measured in triplicate on a Tristar Orion conductivity meter in a conductivity cell 013005MD (Thermo Scientific, Waltham, MA, USA). The apparatus was controlled with the software Navigator 21 (Thermo Scientific) using continuous data collection. The solutions were temperature-controlled to 49 ± 0.1 °C. The surfactant stock solutions had a range of concentrations of 15-200 mM and these stock solutions were diluted further to determine the CMC. A linear pump Lineomat (VEB MLW Labortechnik Ilmenau, Germany) was used with a flow rate of 0.43 mL/min. An AREX stirrer (VELP Scientifica Srl, Usmate, Italy) was used for continuous agitation, and the conductivity was recorded every 3 s. The resulting data were captured into an MS Excel datasheet for subsequent analysis. The transformation of the time axis to a concentration axis was performed according to the parameters defining the measurements (starting volume of a liquid in the titration vessel, the concentration of the surfactant solution in the syringe, and the solution feed rate):

$$C_{t}=\frac{C_{0}}{1+\frac{V_{start}}{v_{inj}*t}}$$

where *C_t_* = concentration at time *t*, *C*_0_ = starting concentration of the surfactant, *V_start_* = volume of distilled water at start, *v_inj_* = surfactant solution dosing rate and *t* = time recorded by Navigator21

### 2.3. Micellar Catalysis

A solution of fenitrothion (O,O-dimethyl O-(3-methyl-4-nitrophenyl) phosphorothioate) was diluted with methanol to obtain a concentration of 10^−2^ M (10 mM). The surfactant stock solutions were diluted in distilled water with a concentration of 500 mM, 100 mM, or 10 mM (pursuant to the value of their CMC). Furthermore, these solutions were diluted in the range of concentrations of 10^−1^–10^−5^ M. Carbonate buffer 0.1 M was adjusted to pH 10 or 11. Carbonate buffer 250 µL, surfactant stock solution 250 µL, and the solution of fenitrothion 10 µL were homogenized in a 1 cm thick cuvette. The absorption changes were measured by Helios α spectrophotometer (Thermo Fisher Scientific) with a set wavelength of 394 nm. The measurement was temperature-controlled to 37 ± 0.1 °C and stopped at the time when the difference between adjacent absorptions was minimal. The resulting data were processed in Thermo Scientific Vision software (Thermo Fisher Scientific) and then converted into MS Excel. The rate constant was calculated by the procedure described by Guggenheim [25], with modification introduced by Zajicek and Radl [26]. The procedure used is based on the acceptance of the following equation:

A_t+∆t_ = A_t_ × exp(k_obs_ × ∆t) + A_n_ ×(1-exp(-k_obs_ × ∆t)),

where A_t+∆t_ = absorption at time t+∆t, A_t_ = absorption at time t, exp = base of natural logarithm, k_obs_ = observed rate constant, ∆t = time between two successive measurements and A_n_ = absorption at infinite time

This relationship is a linear equation with slope exp(k_obs_ × ∆t) and intersection A_n_ × (1-exp(-k_obs_ × ∆t)). Data from ten minutes’ measurement were put in two columns of MS Excel so that the second column was shifted one row up. This shift presents one value ∆t. Data prepared in such a way were displayed in an XY scatter chart. A trends line with a print-out of the coefficient regression equation was interlaid through the displayed points. The slope thus obtained was used for the calculation of the rate constant k_obs_ from the term exp(k_obs_ × ∆t). Rate constants were recalculated to the reaction halftimes [27].

### 2.4. Bacterial Strains

In vitro antibacterial activity of the prepared compounds was tested on a panel of four Gram-positive and four Gram-negative bacterial strains: *Staphylococcus aureus* (C1947), methicillin-resistant *Staphylococcus aureus* (C1926), *Staphylococcus epidermidis* (C1936), vancomycin-resistant *Enterococcus* (S2484), *Escherichia coli* (A1235), extended-spectrum *β*-lactamase (ESBL) non-producing *Klebsiella pneumoniae* (C1950), ESBL-producing *Klebsiella pneumoniae* (C1934), and multi-resistant *Pseudomonas aeruginosa* (A1245). All named bacteria used in the study were obtained as clinical isolates from patients (University Hospital, Hradec Kralove, Czech Republic) and stored at −70 °C in the cryobank according to the manufacturer’s instructions. When needed, each bacterial strain was inoculated and cultivated on Mueller-Hinton agar (HiMedia, Cadersky-Envitek, Prague, Czech Republic). *Francisella tularensis* LVS, live vaccine strain, was obtained from the collection of Faculty of Military Health Sciences, University of Defense (Hradec Kralove, Czech Republic) and cultured on McLeod plates.

### 2.5. Biofilm Cultivation

The second-subculture colonies of *S. aureus* were suspended in Mueller-Hinton broth (MHB) to reach 1.0 McFarland Standard. This suspension was diluted with MHB medium 1:30 and subsequently used as the inoculum. The initial bacterial number of the inoculum was verified by viable cell counting and equaled approximately 10^7^ CFU/mL. Each well from the MBEC microtiter plate (Innovotech, Edmonton, AB, Canada) was inoculated with 150 μL of inoculum. The inoculated device was incubated for 24 h at 35 °C ± 1 °C while being shaken at 110 rpm. For every MBEC assay, the formed biofilm was verified by viable cell counting of disrupted biofilms after removing four pegs from the lid, rinsing and sonication (Bandelin Sonorex, Bandeline Electronics, Berlin, Germany) in MHB for 15 min. The established bacterial number in formed biofilms equals approximately 10^7^ CFU/mL.

### 2.6. Antimicrobial Activity Evaluation

#### 2.6.1. Broth Microdilution Method

The antibacterial susceptibility against bacteria was determined by the broth microdilution method according to standard M07-A10 [28]; the optimized protocol has been published previously [29,30]. Mueller-Hinton broth (Merck, Prague, Czech Republic) adjusted to pH 7.4 (±0.2) was used as the test medium. Dimethyl sulfoxide (DMSO) served as a diluent for all compounds and its final concentration did not exceed 1% in the test medium. The wells of the microdilution tray contained 200 μL of the MH broth with two-fold serial dilutions of the compounds (500–0.49 μmol/L) and were inoculated with 10 μL of the bacterial suspension. The bacterial suspensions were controlled densitometrically to reach 1.5 × 10^8^ viable colony forming units (CFU) per 1 mL. The MIC values, defined as 95% inhibition of bacterial growth, were determined after 24 h and 48 h incubation at 36 °C ± 1 °C. The MBC values were determined as the concentration of compound causing a decrease in the number of bacterial colonies by ≥99.9%, after subculturing of a 10 μL aliquot of each well without visible growth.

#### 2.6.2. Flow Cytometry Assay

*F. tularensis* LVS bacterial suspension in PBS was prepared from fresh overnight subculture to reach O.D. 1.0 (optic density at 600 nm) which in the case of *F. tularensis* means 4 × 10^9^ CFU (colony forming units)/mL. 1 mL aliquots were centrifuged for 7 min, 7000× *g* at 4 °C and the supernatant was replaced by 500 µL of the tested compound. After 5 min exposure the treated bacterial suspension was again centrifuged for 7 min, 7000× *g* at 4 °C and the solution of the tested compound was removed. Cells were re-suspended in 1 mL PBS and stored on ice until flow cytometry measurement. A negative control was prepared from dead bacterial suspension resuspended in PBS (OD 1), where the bacteria had been exposed overnight to 36% formaldehyde. Fresh bacteria cultured overnight on McLeod agar plates were resuspended in PBS (OD 1) and used as a positive control. All samples were diluted 100× in 1 mL of PBS, stained with 1 µL of propidium iodide and 1.5 µL of Syto 9, and incubated in the dark for 15 min. 10 µL of vortexed microspheres were added and samples were properly mixed before measurement. All samples we analyzed with a CyAn ADP Flow Cytometer (Beckman Coulter, Indianapolis, IN, USA). Listmode data were analyzed by Summit v4.3 software (Beckman Coulter). At least three independent experiments were performed.

#### 2.6.3. MBEC Assay

The determination of minimal biofilm eradication concentration (MBEC) against *S. aureus* was determined using MBEC assay plates (Innovotech) according to the manufacturer’s instructions using a modified Calgary method [22]. The biofilm formed on the lid of the MBEC assay microtiter plate was rinsed with saline and inserted on the challenge plate. Each microtiter challenge plate contained tested compounds serially diluted two-fold in MHB, with sterility and growth controls. After incubation for 24 h at 35 °C ± 1 °C, the lid with treated biofilm was removed, rinsed with saline, and the biofilm was disrupted by sonication. Disrupted biofilm was released into a new microtiter plate with MHB supplemented by 0.5% Tween 80 as an antimicrobial neutralizer. The MBEC was determined after 24 h of incubation at 35 °C ± 1° by reading OD_600_. The challenge plate was covered with a new, sterile lid and after 24 h of incubation at 35 °C ± 1 °C was used for MIC determination by reading OD_600_.

### 2.7. Antiviral Activity Evaluation

#### 2.7.1. Viruses and Cell Cultures

Murine cytomegalovirus (MCMV) and SARS-CoV-2 virus were used in the experiments. The viral stocks were prepared by infecting susceptible cells. For MCMV, NIH 3T3 cells (murine embryonic fibroblasts) were used, and for SARS-CoV-2 (clinical isolate kindly provided by Assoc. prof. Daniel Ruzek, University of South Bohemia, Ceske Budejovice, Czech Republic), Vero cells CCL81 (African green monkey kidney) were used.

The viral stocks were prepared after virus inoculation of the appropriate cells in a suitable cell culture medium (DMEM with high glucose, containing 10% fetal calf serum, 2 mM glutamine, 100 U/mL penicillin and 100 µg/mL streptomycin). The cells were incubated at 37 °C under 5% CO_2_ until 80–90% of cells exhibited cytopathic effect (5–7 days). Following 5 min of centrifugation at 500× *g* the supernatant was collected and frozen in aliquots at −80 °C.

#### 2.7.2. Virucidal Activity Testing

The virucidal activity of the synthesized agents was evaluated by a quantitative suspension test. Briefly, one part of the virus suspension was mixed with one part of bovine serum albumin (final concentration 0.03%; ‘clean conditions’ as specified in EN 14476) and eight parts of the novel tested compounds. The mixtures were kept at room temperature for 5 min (exposure time). After the exposure period, the mixtures were serially diluted ten-fold with ice-cold DMEM and 100 µL of each dilution were seeded into a 96-well microtiter plate with the appropriate cell and incubated at 37 °C in a 5% CO_2_ atmosphere until a cytopathic effect was detected, approximately 5–7 days. Six wells per sample dilution were inoculated. Virus-untreated controls with identical protein concentrations were also tested.

Virus titer was determined using the method of Spearman and Karber and expressed as log_10_ TCID_50_/mL [31,32]. According to the European Standard EN 14476 for the virucidal activity of disinfection, a product should demonstrate at least a log_10_ reduction of 4 in virus titer, corresponding to 99.99% inactivation [33]. To differentiate between virus-induced cytopathogenic changes and the toxic effect caused by the tested compounds, the cell monolayer was monitored until the end of the virucidal activity testing period for morphological changes after exposure to the disinfectant solution.

### 2.8. In Vitro Cytotoxicity and Selectivity Index

The standard 3-(4,5-dimethylthiazol-2-yl)-2,5-diphenyltetrazolium bromide (MTT, Merck) cell viability assay was applied according to the manufacturer’s protocol on the Chinese hamster ovary cell line CHO-K1 (ECACC, Salisbury, UK) [34]. The cells were cultured according to ECACC recommended conditions and seeded at a density of 8 × 10^3^ per well in the 96-well plate. The tested compounds were dissolved in DMSO (Merck,) and subsequently in the Nutrient Mixture F-12 Ham growth medium (Merck) supplemented with 10% Fetal Bovine Serum and 1% Penicillin-Streptomycin (both Merck) so that the final concentration of DMSO did not exceed 1% (*v/v*) per well. Cells were exposed to the tested compounds for 24 h. The medium was replaced by a medium containing 0.5 mg/mL of MTT and the cells were allowed to produce formazan for approximately 3 h under surveillance. Thereafter, the medium with MTT was removed and the crystals of formazan were dissolved in DMSO (100 µL/well; Ing. Petr Švec–PENTA s.r.o., Prague, Czech Republic). Cell viability was assessed spectrophotometrically by the amount of formazan produced. Absorbance was measured at 570 nm with 650 nm as a reference wavelength on Synergy HT (BioTek, Winooski, VT, USA). The IC_50_ value was then calculated from the control-subtracted triplicates using non-linear regression (four parameters) by GraphPad Prism 5.03 or 7.03 software (GraphPad Software, Inc., San Diego, CA, USA). Final IC_50_ values including SEM (standard error of the mean) were obtained as the mean of three independent measurements. The selectivity indices of compounds have been calculated as the ration of the cytotoxicity (IC_50_) and minimum inhibitory concentrations after 24 h (MIC) for all bacterial strains.

## 3. Results and Discussion

### 3.1. Synthesis

**Scheme 1:**
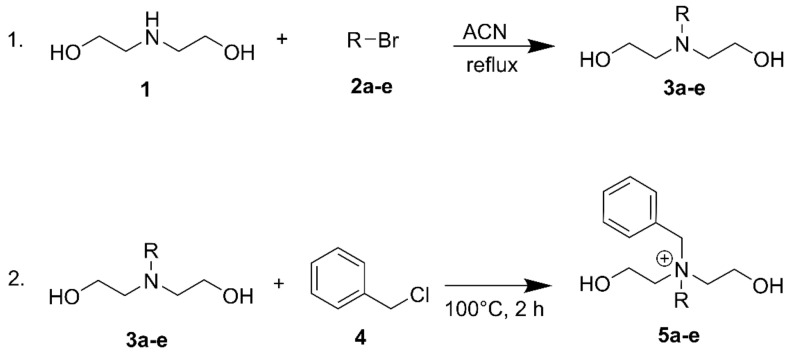
Two-step preparation of benzoxonium-like salts.

The series of alkyl-bis(2-hydroxyethyl)benzylammonium chlorides **5a-e** was prepared by the two-step reaction according to Limanov et al. [35]. The reaction process is shown in Scheme 1.

Yields, melting points, purity, and Clog *P* are listed in Table 1. Compound **5b** was prepared with a superior yield compared to Chernyavskaya et al. [36], but with inferior yield according to Limanov et al. [35] prepared in the same way. Homologues **5a** and **5d** were obtained in a yield comparable to that of Stefanovic et al. [37]. The decreased yields are the result of repeated crystallizations to reach satisfactory purity of the final products. Compounds **5c** and **5e** were reported for the first time.

### 3.2. Conductometry

**Figure 1:**
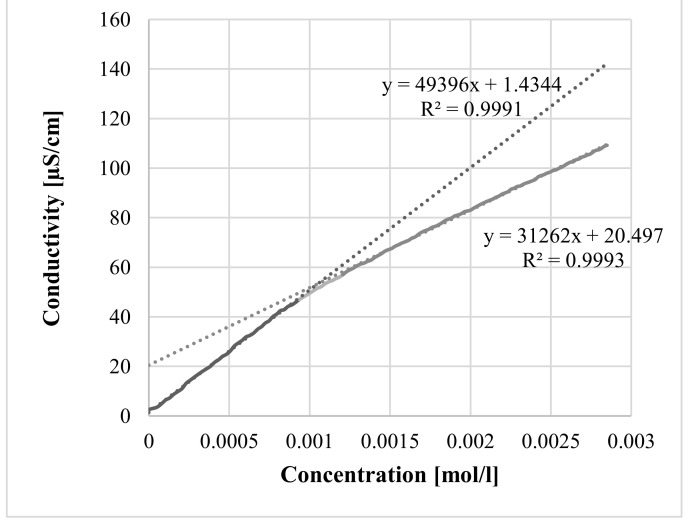
Dependency of conductivity on concentration. The curve was divided into three parts: lower and upper linear parts, and a transition section. The equation of the line and the coefficient of determination were calculated for both linear parts. The CMC value was determined at the intersection of both axes [38].

The critical micelle concentrations (CMC) of the new series of compounds were assessed by a conductometric study performed in aqueous solutions at 49 °C, which takes advantage of the dependency between conductivity change and rising concentration of surfactant. The dependence curve is linear until the unimers of the surfactants can balance the interactions between solution and micelle aggregation. This change is manifested as a breaking of the curve as shown in Figure 1. 

**Figure 2:**
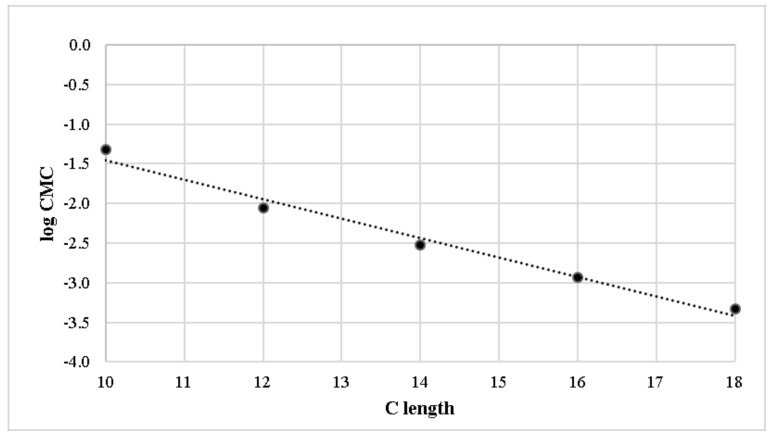
Dependency of log CMC on length of alkyl chain.

According to Taube’s rule [39], obtained data confirm the dependency of CMC on the elongation of the alkyl chain (Figure 2). The CMC values (Table 2) are the basic characteristics of surfactants needed for further chemical decontamination measurement.

### 3.3. Micellar Catalysis

Next, the hydrolytic activity against fenitrothion was measured. Because the pesticide fenitrothion contains a 4-nitrophenol group detectable by UV/VIS spectrophotometry, it is a suitable candidate for micellar catalysis investigation as a representative of highly toxic organophosphorus compounds with chemical warfare potential [40]. Reaction of fenitrothion with a micellar catalyst provides 3-methyl-4-nitrophenol, which in alkaline medium creates the yellow-colored nitrophenoxide anion with absorption maximum at 400 nm [27]. This decomposition reaction is monitored by the spectrophotometer. Hydrolytic activity was evaluated in this way using the Guggenheim method for the whole series of synthesized compounds **5a-e** as an assessment of chemical decontamination potential (Table 3). This method is applicable in the case of first order or pseudo-first order reactions and enables processing of the data using linear regression [25]. Compound **5d** is the most efficient micellar catalyst with half-life 2.3 min at 0.01M concentration, and half-life 2.4 min at 0.005M (pH 11 and 37 °C). In comparison with the spontaneous hydrolysis of fenitrothion (k = 14.00 × ^−6^, T_1/2_ = 808.4 min) under the same conditions, the novel compounds were 60–160 times more effective at pH 11. It is evident that increasing the pH by one unit causes a significant acceleration of the organophosphate degradation. Spontaneous hydrolysis of fenitrothion at pH 10 was so slow that it could not be determined by the given methodology.

The rate constants determined in the concentrations below the CMC values were affected by spontaneous hydrolysis at a given temperature, by pH, and the presence of hydroxyl groups hydrolyzing the fenitrothion ester bond. Once the concentration exceeds the CMC, the rate constant noticeably rises. Hydroxyl groups and formed micelles together create a counterion cationic surfactant, and fenitrothion is attracted to the Stern layer where it adopts a favorable orientation for nucleophile access and is finally decomposed [41].

**Figure 3:**
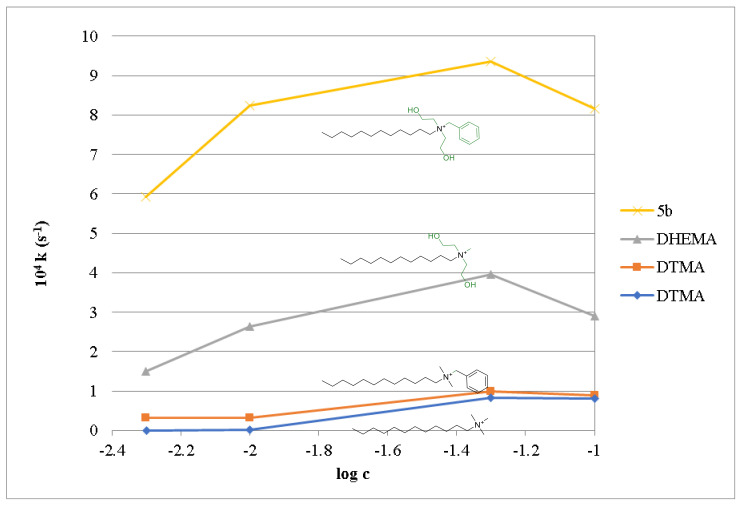
Commercially used compounds at pH 11. The influence of QAS structure on the decontamination effect (rate constant) at pH 11; DTMA (*N*-dodecyl-*N*,*N*,*N*-trimethylammonium chloride), BAC (*N*-benzyl-*N*,*N*-dimethyl-*N*-dodecylammonium chloride), DHEMA (*N,N*-bis(hydroxyethyl)-*N*-dodecyl-*N*-methylammonium chloride) and **5b** (*N*-benzyl-*N*,*N*-bis(2-hydroxyethyl)dodecan-1-aminium chloride; green color highlights the positive moiety to increase the decontamination effect. The decrease in activity at high concentrations is due to an excess of surfactant. This so-called empty micelle effect has been previously described [42].

The structure-activity relationship evaluation showed a clear effect of the presence of the hydroxyethyl groups on the rate constant of OP decomposition. Indeed, Figure 3 shows asymptotic dependence between rate constant and concentration for the commercially used compounds with identical length of alkyl chain C_12_ at pH 11. Whereas DTMA (*N*-dodecyl-*N*,*N*,*N*-trimethylammonium chloride) and BAC (*N*-benzyl-*N*,*N*-dimethyl-*N*-dodecylammonium chloride), commercially used QAS without hydroxyethyl groups, show poor decontamination effect, DHEMA (*N,N*-bis(hydroxyethyl)-*N*-dodecyl-*N*-methylammonium chloride) containing hydroxyethyl groups shows a significant increase in decontamination effect. Notably, 5**b** was found to be much superior to all the reference compounds. Finally, we proved the necessity for the hydroxyl groups potentiated by the benzyl moiety for justification of the choice of BOC-like compounds.

### 3.4. Antimicrobial Activity

**Figure 4:**
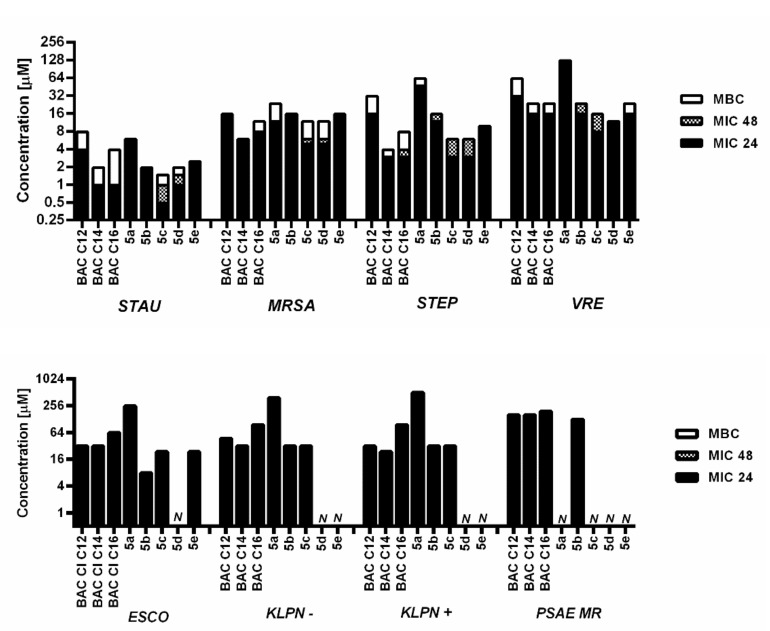
MIC and MBC values of tested compounds for G+ and G- bacterial strains. MIC values were determined after 24 and 48 h of incubation, MBC was determined after 24 h of incubation. The results for BACs had been published elsewhere [31,38]. *N* means that MIC or MBC was higher than the highest soluble concentration of the compound. Abbreviations: STAU = *S. aureus*, MRSA = methicillin-resistant *S. aureus*, STEP = *S. epidermidis*, VRE = vancomycin-resistant *Enterococcus,* ESCO = *E. coli,* KLPN- = *K. pneumoniae,* KLPN+ = extended-spectrum *β*-lactamase-producing *K. pneumoniae*, PSAE MR = multidrug-resistant *P. aeruginosa*.

To evaluate the biological decontamination potential, in vitro antimicrobial activity of compounds **5a-e** was evaluated by the broth microdilution method for four Gram-positive and four Gram-negative bacterial strains. Obtained results are presented as minimum inhibitory concentrations (MICs) after 24 h and 48 h, and the minimum bactericidal concentrations (MBCs) after 24 h (Figure 4). High antibacterial activity was found against the G+ bacteria *S. aureus* (STAU), methicillin-resistant *S. aureus* (MRSA), *S. epidermidis* (STEP), and vancomycin-resistant *Enterococcus* (VRE). The observed higher susceptibility to the tested compounds of G+ bacteria compared to G- bacteria, as discussed previously, is in agreement with the literature [43,44]. The lowest MIC and MBC values, i.e., the highest effectiveness, was observed for *S. aureus*. The relationship of alkyl chain length to antimicrobial activity showed that the compounds with C_14_ (**5c**) and C_16_ (**5d**) chains were the most efficient against G+ bacteria. In contrast, the G- bacteria *E. coli* (ESCO)*, K. pneumoniae* (KLPN-)*,* extended-spectrum *β*-lactamase-producing *K. pneumoniae* (KLPN+), and multidrug-resistant *P. aeruginosa* (PSAE MR) were most susceptible to compounds with shorter alkyl chains, C_12_ (**5b**) and C_14_ (**5c**), respectively. Unsurprisingly, the least efficient against all tested bacterial strains was the compound with the shortest alkyl chain C_10_ (**5a**) [14,29,31]. Compared with the commercially used BAC series, the **5a-e** series was comparable at a corresponding alkyl chain length. Individually, **5c** and **5b** have shown the best activity against *S. aureus* and *E. coli,* respectively.

**Figure 5:**
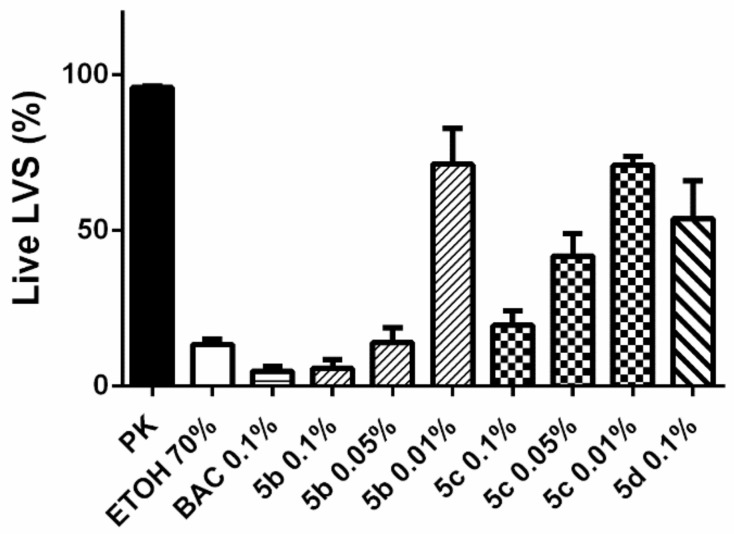
Susceptibility testing against *Francisella tularensis* LVS. The remaining ratio of living LVS after 5 min exposure is expressed as mean ± SEM (*n* = 3).

**Figure 6:**
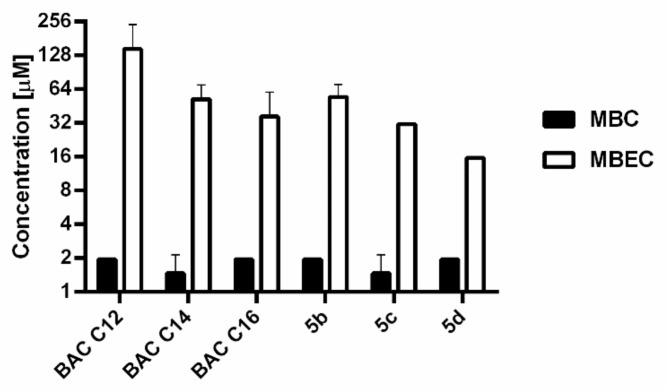
MBEC values and MBC values established for *S. aureus.* MBC and MBEC values were determined after 24 h of incubation for BAC and **5b-d** series by broth microdilution method and MBEC assay respectively. Results are expressed as the mean ± SD (*n* = 3).

According to the MICs and MBCs obtained for the traditional strains, the most efficient derivatives (**5b**, **5c**, and **5d**) underwent susceptibility testing against *Francisella tularensis* Live Vaccine Strain (LVS) by flow cytometry assay (Figure 5). Although we used a *F. tularensis* LVS model strain that is attenuated in comparison to the virulent strain, the resistance of this strain is identical to that of the virulent strains. *F. tularensis* is a Gram-negative, intracellular bacterium which is an important medical challenge. *F. tularensis* is included in category A on the Centers for Disease Control and Prevention (CDC) bioterrorism agent/disease list for the following reasons [45]: (a) it can be easily disseminated or transmitted from person to person; (b) it results in high mortality rates and has the potential for major public health impact; (c) it might cause public panic and social disruption; and (d) it requires special action for public health preparedness. In view of the above, any substance that can successfully kill *F. tularensis* is highly desirable. The time of exposure (5 min) and reference compounds (ethanol 70% and BAC 0.1%) were used to simulate more practical conditions. The obtained results showed high efficacy especially of **5b,** at least comparable to all reference drugs (Figure 6). Notably, substance **5b** at concentration 0.1% has an even better effect compared to ethanol, which is used as standard for disinfection and decontamination against pathogenic bacteria. Moreover, the effect of alkyl chain length on susceptibility to G- bacteria, represented by decreasing activity from **5b** to **5d**, corresponded to results obtained by microdilution assay. In addition, it is clear that the decontamination effect is proportionally dependent on the concentration (**5c** at concentration of 0.1%, 0.05% and 0.01% causes the elimination of 80.47 ± 4.61%, 58.16 ± 7.23% and 29.04 ± 8.80% of bacteria, respectively) (Figure 5).

### 3.5. Antibiofilm Activity and Comparison in Effectiveness against S. aureus in Planktonic and Biofilm Form

To evaluate the efficacy of the novel series against the biofilm form, which is much more resistant than the planktonic form and can often cause a relapse of the infection, a S. aureus biofilm formed on a polystyrene surface for 24 h was exposed for 24 h to the 5b, 5c, 5d and BAC derivatives with C12, C14 and C16 alkyl chains. The minimal biofilm eradication concentration (MBEC) showed comparable activity between both tested series, and notably the MBECs values of 5b-d derivatives were slightly lower than the values for the corresponding BAC derivatives. Comparison of established MBEC and MBC values have shown that S. aureus in biofilm form was 4–64 times less susceptible to the tested compounds than in planktonic form. All results are depicted in Figure 6.

### 3.6. Virucidal Activity against Enveloped Viruses

To evaluate the width of the spectrum against different microorganisms, the virucidal activity of **5b-d** on murine cytomegalovirus (MCMV) and SARS-CoV-2 virus was determined using a quantitative suspension assay. Virus titer was evaluated using the Spearman-Karber method and expressed as logTCID50/mL [32]. In accordance with EN 14476 for disinfection with full virucidal activity, the substance should demonstrate a reduction in virus titer of at least 4 orders of magnitude, corresponding to 99.99% inactivation [33]. The morphological changes of one layer of cells were monitored to distinguish between the virus-induced cytopathic changes and the toxic effect caused by the test substance. The highest efficiency was shown by substance **5b**, where the virus titer decreased by more than 5 orders of magnitude at a concentration of 0.01% (Table 4). The decrease could not be determined exactly for the compounds at a concentration of 0.1% (marked>), as the tested substances at this concentration already affected the cell culture which was used for growing the virus. Evaluation against SARS-CoV-2 showed that **5b** and **5c** showed only a modest effect at a concentration of 0.01%. Unfortunately, at higher concentration (0.1%), growth of the cells for culturing the virus was affected and the effect reaching the required value of a decrease of 4 logarithms cannot be confirmed.

### 3.7. Cell Viability Evaluation and Selectivity Index

To define the cytotoxic selectivity, i.e., safety, towards eukaryotic cells, in vitro cytotoxicity studies were performed using the mammalian Chinese hamster ovary (CHO)-K1 cell line. The values of IC_50_ (half maximal inhibitory concentration) confirmed the predicted trend of the correlation described earlier for the homolog group, i.e., elongation of the alkyl chain length increases the cytotoxic potential of the drugs (Table 5) [29,31]. This effect is related to the increasing lipophilicity expressed as the Clog *P* (Table 5), and most likely correlates with the easier ability to penetrate cells. Notably, considering the identical BAC homologs (BAC 12-16), we can conclude that the introduction of the hydroxyethyl groups into the structure increases the IC_50_ value and therefore reduces the toxicity of the substances. Furthermore, the selectivity indexes were calculated as the ration of IC_50_/MIC (24 h) for all bacterial strains (Table 6).

## 4. Conclusions

The series of benzoxonium-like salts with alkyl chain length C_10_, C_12_, C_14_, C_16_ and C_18_ was identified as promising active ingredients for chemical-biological decontamination. Critical micellar concentration as a standard characteristic of surfactants has shown a suitable concentration for chemical decontamination and the efficacy in fenitrothion degradation surpassed that of all the reference compounds. Biocidal activity against various strains of bacteria including *F. tularensis* and *S. aureus* in biofilm form was also determined to discover biological decontamination potential. The homolog with a chain length of C_12_ (**5b**) shows the highest biocidal activity. However, the best effect for chemical decontamination is observed for derivatives with a longer alkyl chain (i.e., compounds **5d**, **5e**). In addition, the compounds seem to be safe on mammalian cell lines as a basic safety parameter for potential use in practice. Finally, BOC-type compounds have been found to have a significant dual effect against both chemical and biological agents. It is thus possible to envisage their use, individually or in a mixture, as active components of multi-purpose decontamination products.

## Figures and Tables

**Table 1:** Synthesis of BOC-like salts.

Compound	R	Yield (%)	m.p. (°C)	Purity (%)	Clog *P*
**5a**	C_10_H_21_	79	114.0–114.6	97	−1.907
**5b**	C_12_H_25_	42	118.7–119.0	95	−1.018
**5c**	C_14_H_29_	67	120.6–120.9	95	−0.129
**5d**	C_16_H_33_	69	121.6–121.9	98	0.76
**5e**	C_18_H_37_	7	120.6–122.3	95	1.649

**Table 2:** CMC value of compounds **5a-5e**.

C Length (Compound)	CMC [mol/L]	log CMC
10 (**5a**)	4.836 × 10^−2^	−1.316
12 (**5b**)	8.710 × 10^−3^	−2.060
14 (**5c**)	3.003 × 10^−3^	−2.522
16 (**5d**)	1.159 × 10^−3^	−2.936
18 (**5e**)	4.707 × 10^−4^	−3.332E

**Table 3:** Summary data of fenitrothion degradation.

Compound	c (mM)	pH 11, t 37 °C		pH 10, t 37 °C	
		10^4^ k (s^−1^)	T_1/2_ (min)	10^4^ k (s^−1^)	T_1/2_ (min)
**5a**	500	6.20	18.6	2.30	50.3
	100	12.82	9.0	3.97	29.2
	50	12.68	9.1	3.77	30.7
	10	0.50	230.8	0.18	606.3
	5	0.35	323.2	-	-
**5b**	100	8.17	14.1	1.67	69.1
	50	9.35	12.4	2.35	49.3
	10	8.23	14.0	2.65	43.5
	5	5.93	19.5	1.15	100.8
	1	1.80	64.5	-	-
**5c**	10	**24.62**	**4.7**	6.77	17.1
	5	**23.62**	**4.9**	5.82	19.9
	1	11.28	10.2	2.02	57.5
	0.5	3.80	30.5	1.12	103
	0.1	1.17	98.8	0.50	230.8
**5d**	10	**50.73**	**2.3**	11.56	10.0
	5	**49.06**	**2.4**	11.14	10.4
	1	12.32	9.4	3.97	29.2
	0.5	6.47	17.9	2.30	50.3
	0.1	1.77	65.3	1.10	105.2
**5e**	5	23.30	5.0	6.98	16.5
	1	13.95	8.3	4.37	26.4
	0.5	7.82	14.8	2.97	38.9
	0.1	1.87	62.0	0.82	142.5
	0.05	0.83	138.4	0.28	404.1
**SH**		0.14	808.4	-	-

(–) values were outside the instrumental range. Concentration (c), half-life (T_1/2_), and rate constant (k). Left column includes measurement at pH 11 and 37 °C and right column includes measurement at pH 10 and 37 °C. The compounds are sorted in descending effect as follows **5d** > **5c** > **5e** > **5a** > **5b**. Spontaneous hydrolysis (SH) of fenitrothion at pH 11–T_1/2_ = 808.4 min; at pH 10 it could not be determined by given methodology.

**Table 4:** Determination of virucidal activity against MCMV and SARS-CoV-2 (5 min exposure).

Compounds	Conc. (mM)	ΔlogTCID50 (MCMV)	ΔlogTCID50 (SARS-CoV-2)
**5b**	0.1% (2.690)	>3.34 ^a^	>3.5 1^a^
	0.01% (0.269)	5.33	0.47
**5c**	0.1% (2.340)	>2.34 ^a^	>3.51 ^a^
	0.01% (0.234)	3.34	0.64
**5d**	0.1% (2.190)	>3.34 ^a^	1.00
	0.01% (0.219)	−0.50	0.84

^a^ The symbol > indicates that the concentration of the tested substances already affected the cell culture used for virus cultivation and it is not possible to determine the exact value.

**Table 5:** The effect of the tested compounds on CHO-K1 cell viability. Values are expressed as the IC_50_ (series **5a-e**): Mean ± SEM (*n* = 3).

Compound	IC_50_ ± SEM (µM)	Clog *P*
**5a**	128.40 ± 12.7	−1.91
**5b**	36.09 ± 0.5	−1.02
**5c**	27.34 ± 1.1	−0.13
**5d**	19.58 ± 0.5	0.76
**5e**	19.14 ± 1.3	1.64
**BAC 12 ^a^**	19.54 ± 1.2	2.63
**BAC 14 ^a^**	15.04 ± 0.1	3.52
**BAC 16 ^a^**	12.85 ± 1.4	4.41

^a^ The preparation of **BAC_12-16_** has been described elsewhere [46].

**Table 6:** The selectivity indexes of benzoxonium-like salts calculated as ration IC_50_/MIC (24h).

	SI (IC_50_/MIC)							
Compounds	STAU	MRSA	STEP	VRE	ESCO	KLPN-	KLPN+	PSAE MR
**5a**	21.986	10.956	2.739	1.027	0.514	0.342	0.257	<0.257
**5b**	18.508	2.309	3.079	2.309	4.621	1.155	1.155	0.289
**5c**	55.796	5.602	9.331	3.501	1.166	0.875	0.875	<0.437
**5d**	19.980	4.012	6.683	1.671	<0.313	<0.313	<0.313	<0.313
**5e**	8.700	1.225	1.959	1.225	0.817	<0.612	<0.612	<0.612
**BAC 12**	3.750	0.625	0.625	0.375	0.313	0.156	0.094	0.039
**BAC 14**	13.193	5.127	3.847	2.310	0.722	0.577	0.241	0.030
**BAC 16**	19.668	0.987	4.936	3.946	1.234	0.822	0.822	<0.051

Abbreviations: STAU = *S. aureus*, MRSA = methicillin-resistant *S. aureus*, STEP = *S. epidermidis*, VRE = vancomycin-resistant *Enterococcus,* ESCO = *E. coli,* KLPN- = *K. pneumoniae,* KLPN+ = extended-spectrum *β*-lactamase-producing *K. pneumoniae*, PSAE MR = multidrug-resistant *P. aeruginosa,* SI—selectivity index, MIC—minimum inhibitory concentration (24 h).

## Data Availability

Not applicable.

## Supplementary Materials

The following are available online at https://www.mdpi.com/article/10.3390/toxics9090222/s1, Figure S1: Figure S1: ^1^H NMR (500 MHz, Chloroform-*d*) – 5a, Figure S2: 13C NMR (126 MHz, Chloroform-d) – 5a, Figure S3: 1H NMR (500 MHz, Chloroform-d) – 5b, Figure S4: 13C NMR (126 MHz, Chloroform-d) – 5b, Figure S5: ^1^H NMR (500 MHz, Methanol-*d_4_*) – 5c, Figure S6: ^13^C NMR (126 MHz, Methanol-*d_4_*) – 5c, Figure S7: ^1^H NMR (500 MHz, Chloroform-*d*) – 5d, Figure S8: ^13^C NMR (126 MHz, Chloroform-*d*) – 5d, Figure S9: ^1^H NMR (500 MHz, Chloroform-*d*) – 5e, Figure S10: ^13^C NMR (126 MHz, Chloroform-d) – 5e, Figure S11: MS spectrum of 5a in Rt 3.15 min, Figure S12: MS spectrum of 5b in R_t_ 3.28 min, Figure S13: MS spectrum of 5c in Rt 3.46 min, Figure S14: MS spectrum of 5d in Rt 3.64 min, Figure S15: MS spectrum of 5e in Rt 3.84 min.
