# Supplementary material for: Synthesis and Decontamination Effect on Chemical and Biological Agents of Benzoxonium-Like Salts
Source: Toxics. 2021 Sep 15;9(9):222. doi: 10.3390/toxics9090222 (PMC8469817; doi:10.3390/toxics9090222)
Supplement: Supplementary file 1 [file toxics-09-00222-s001.zip › toxics-1338237-supplementary.pdf]

Supplementary Materials: Synthesis and decontamination effect on chemical and biological agents of benzoxonium-like salts.

Aneta Markova, Michaela Hympanova, Marek Matula, Lukas Prchal, Radek Sleha, Marketa Benkova, Lenka Pulkrabkova, Ondrej Soukup, Zuzana Krocova, Daniel Jun and Jan Marek

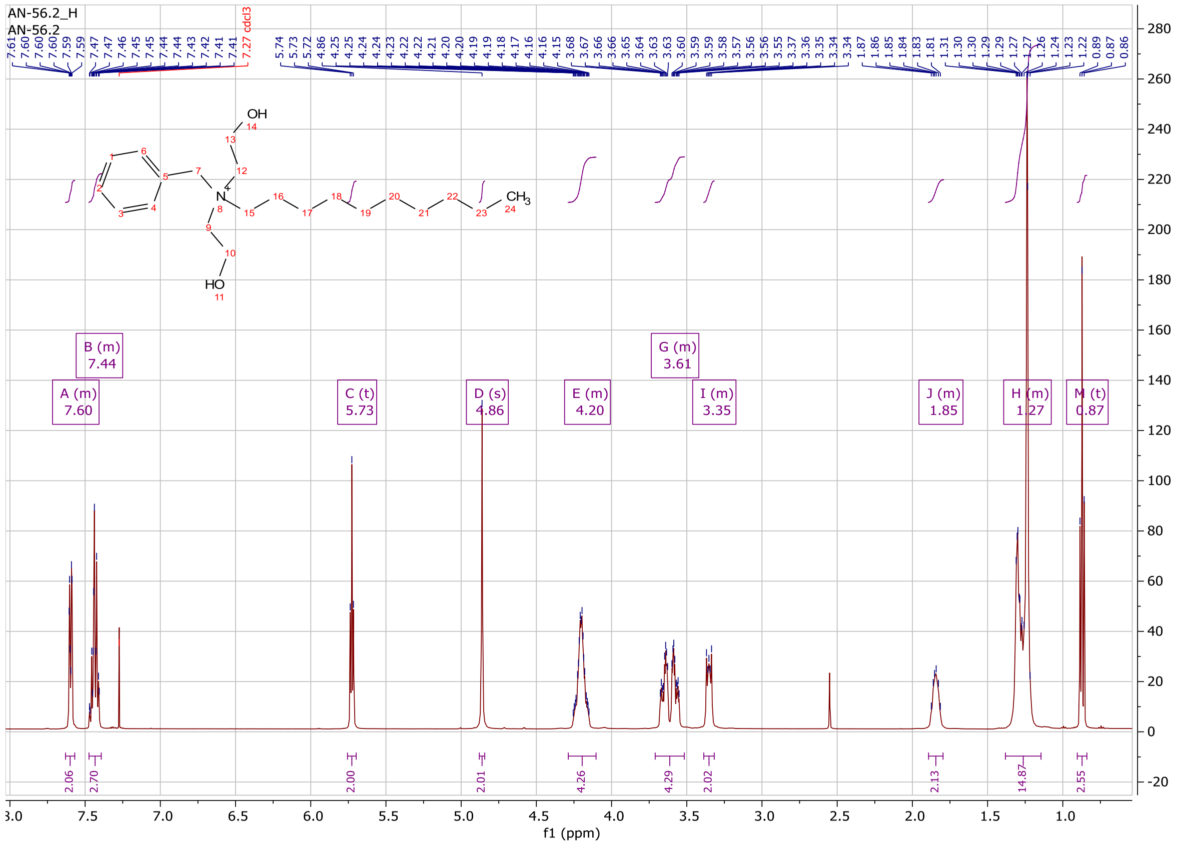

Figure S1. ^{1}H NMR (500 MHz, Chloroform-*d*) – **5a**

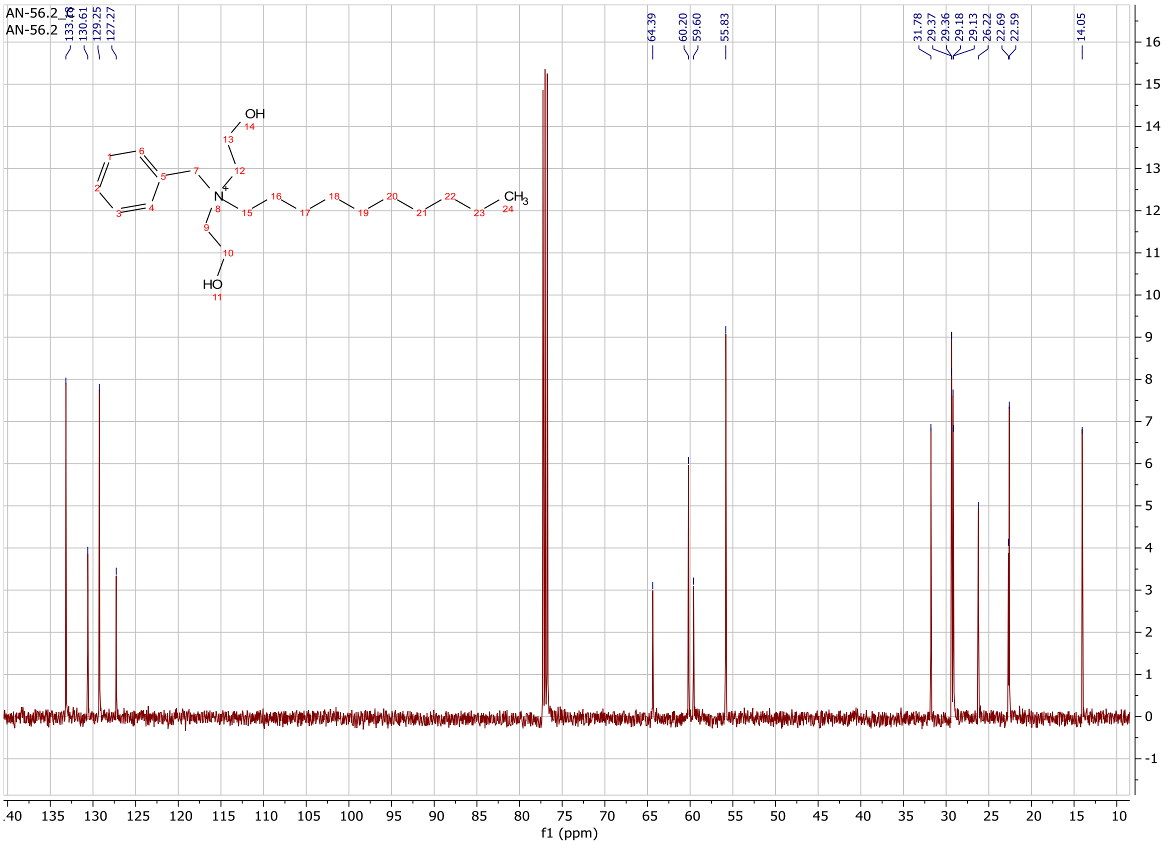

Figure S2. ^{13}C NMR (126 MHz, Chloroform-*d*) – **5a**

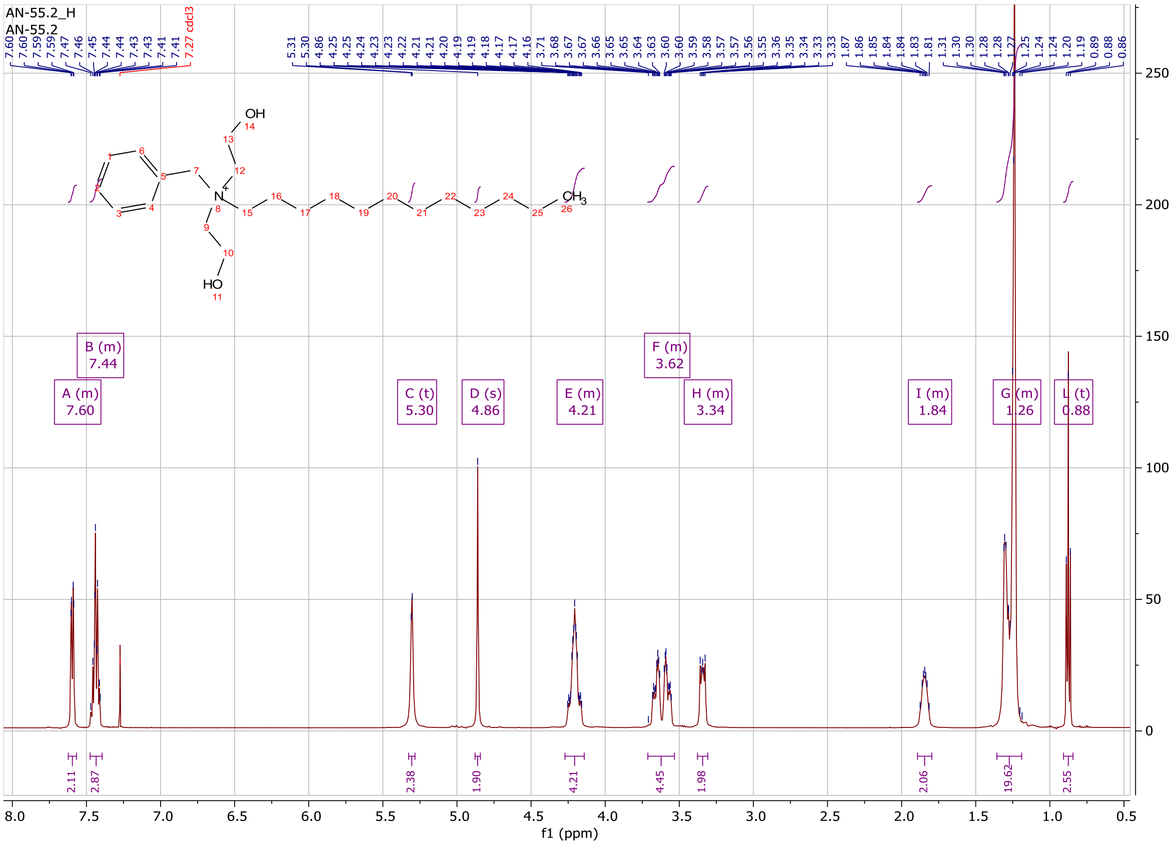

Figure S3. ^{1}H NMR (500 MHz, Chloroform-*d*) – **5b**

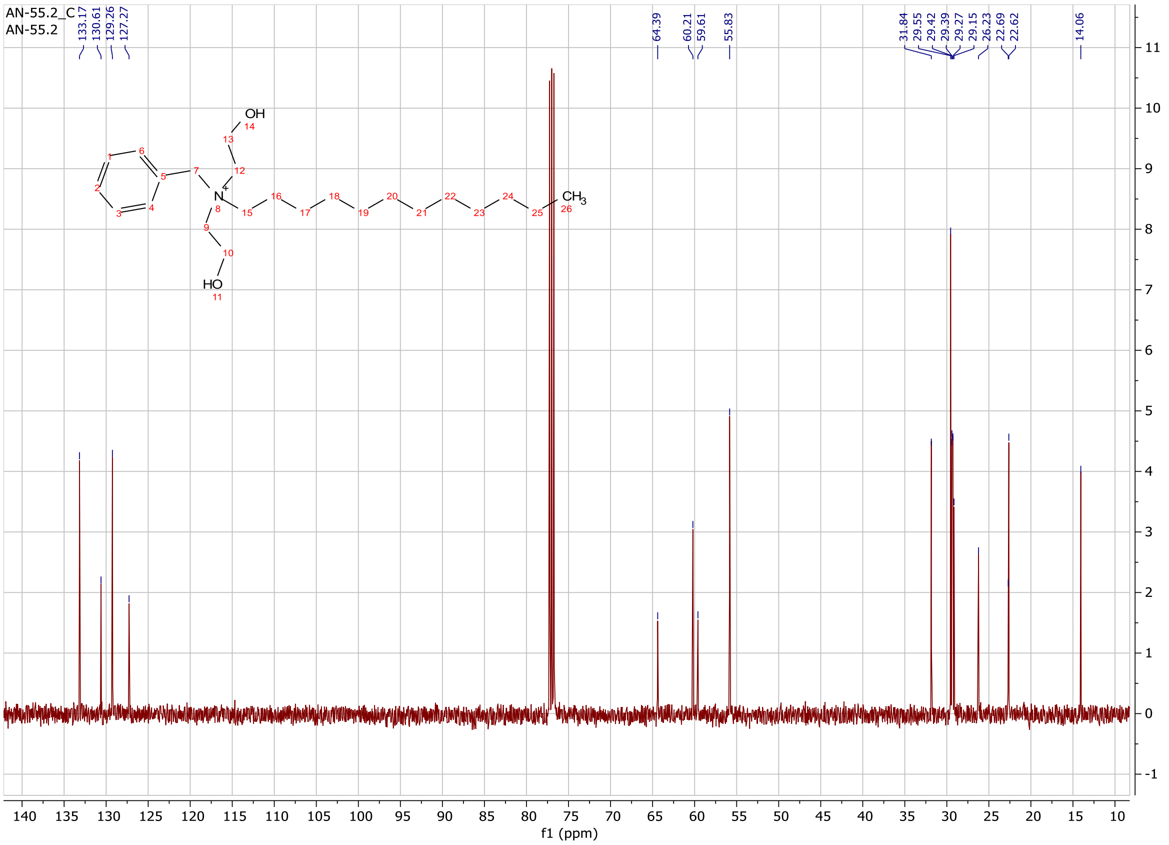

Figure S4. ^{13}C NMR (126 MHz, Chloroform-*d*) – **5b**

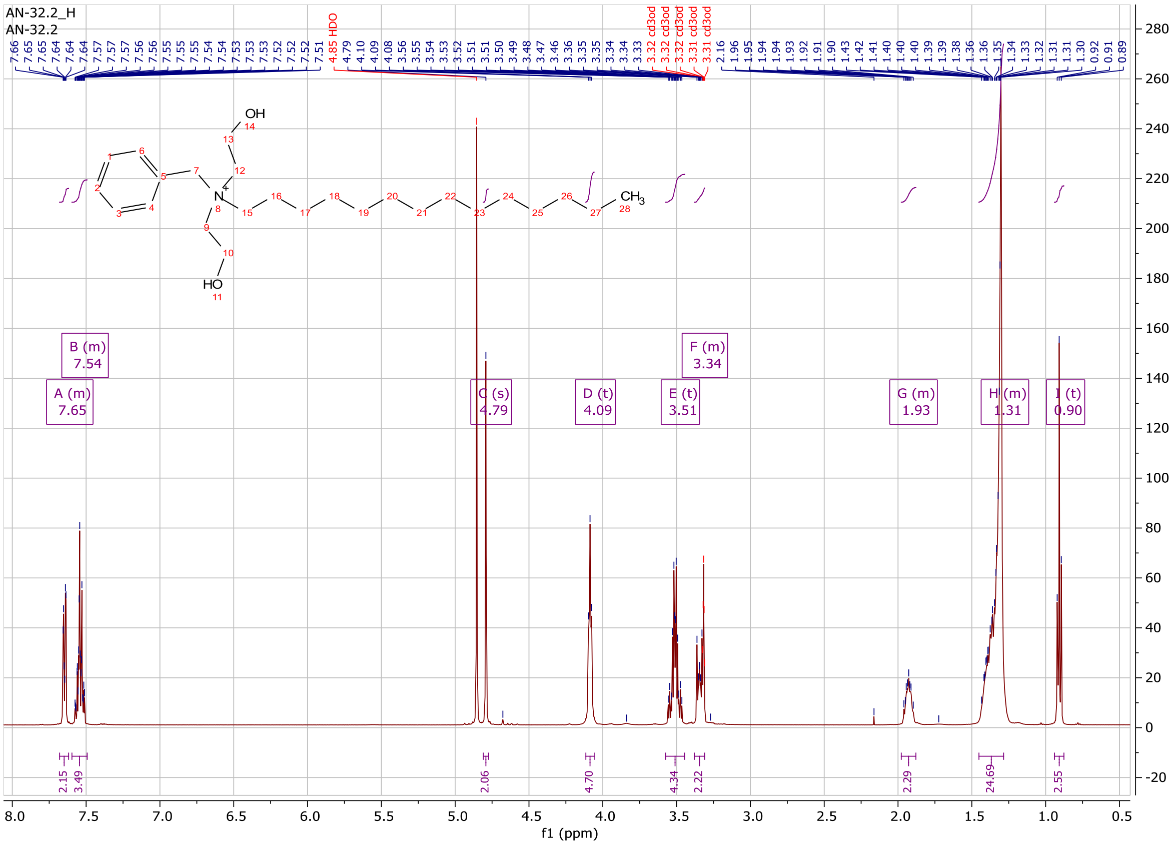

Figure S5. ^{1}H NMR (500 MHz, Methanol-*d*$_4$) – **5c**

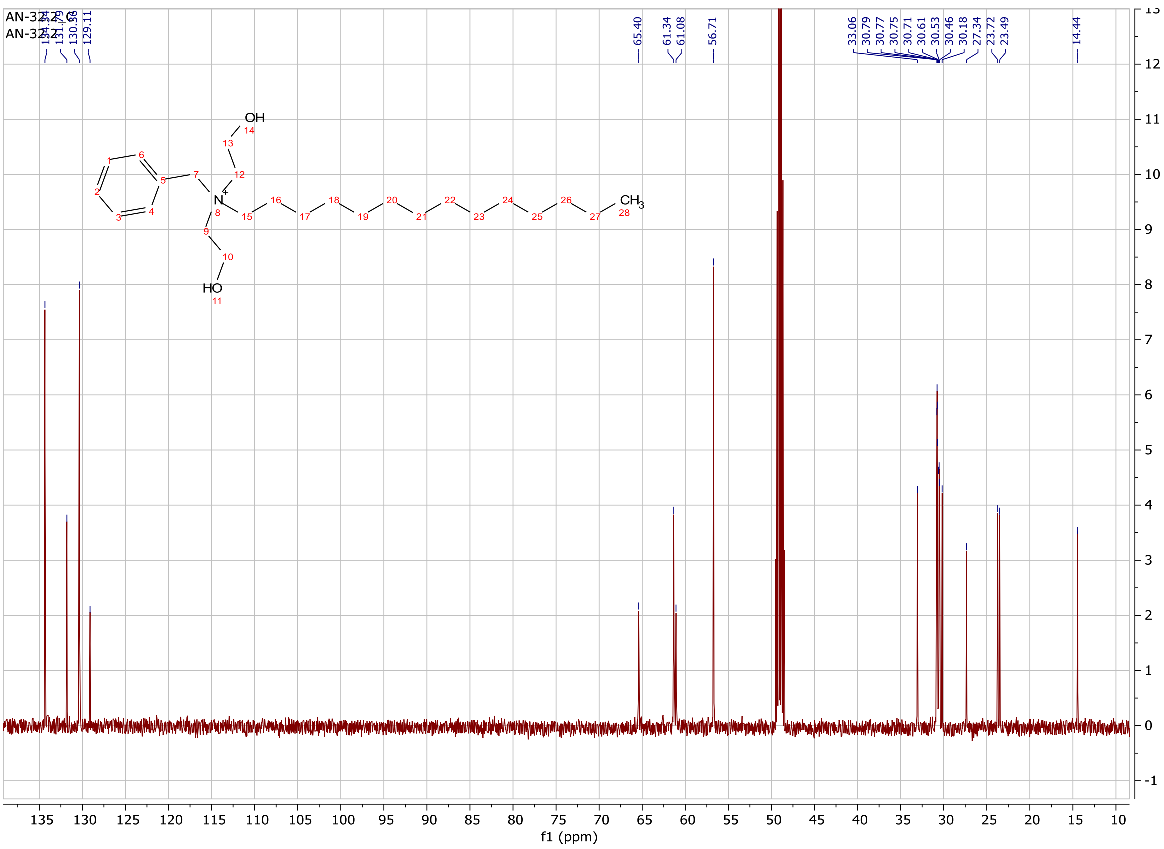

Figure S6: ^{13}C NMR (126 MHz, Methanol-d_4) – **5c**

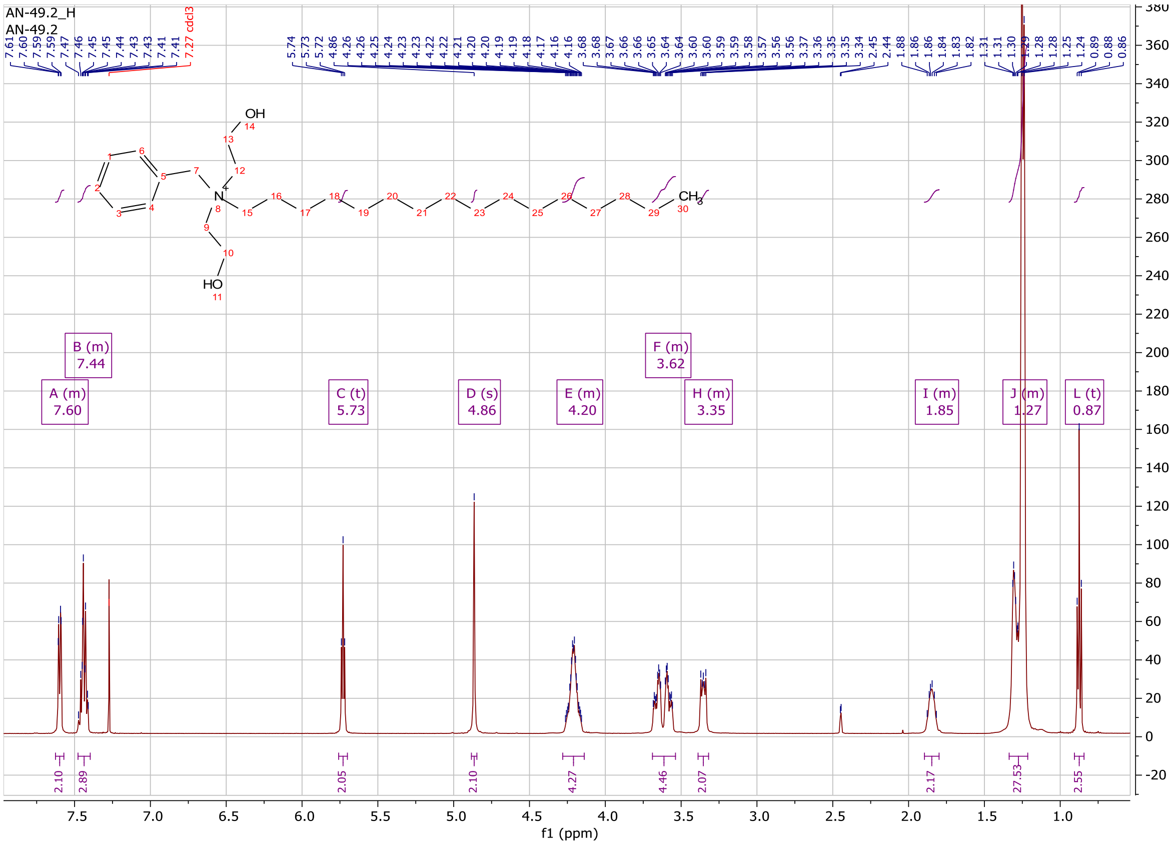

Figure S7. ^{1}H NMR (500 MHz, Chloroform-*d*) – **5d**

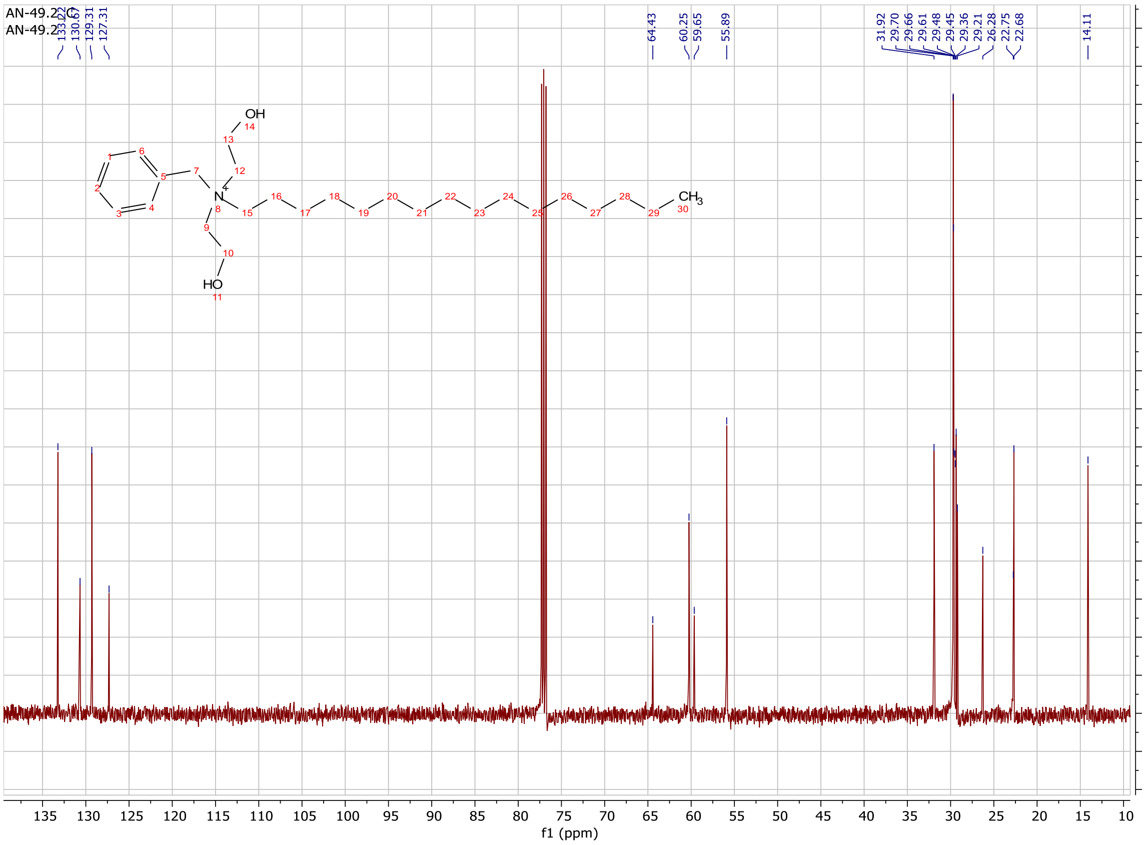

Figure S8. ^{13}C NMR (126 MHz, Chloroform-*d*) – **5d**

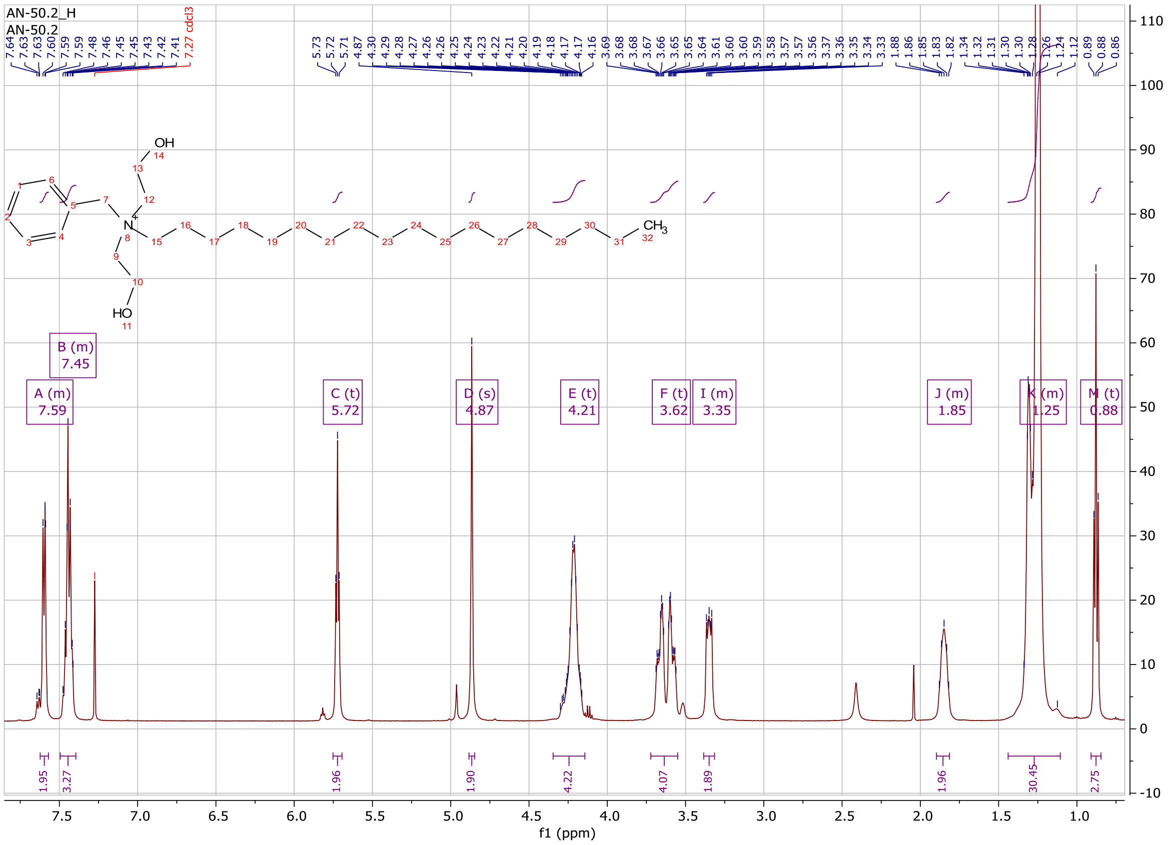

Figure S9. ^{1}H NMR (500 MHz, Chloroform-*d*) – **5e**

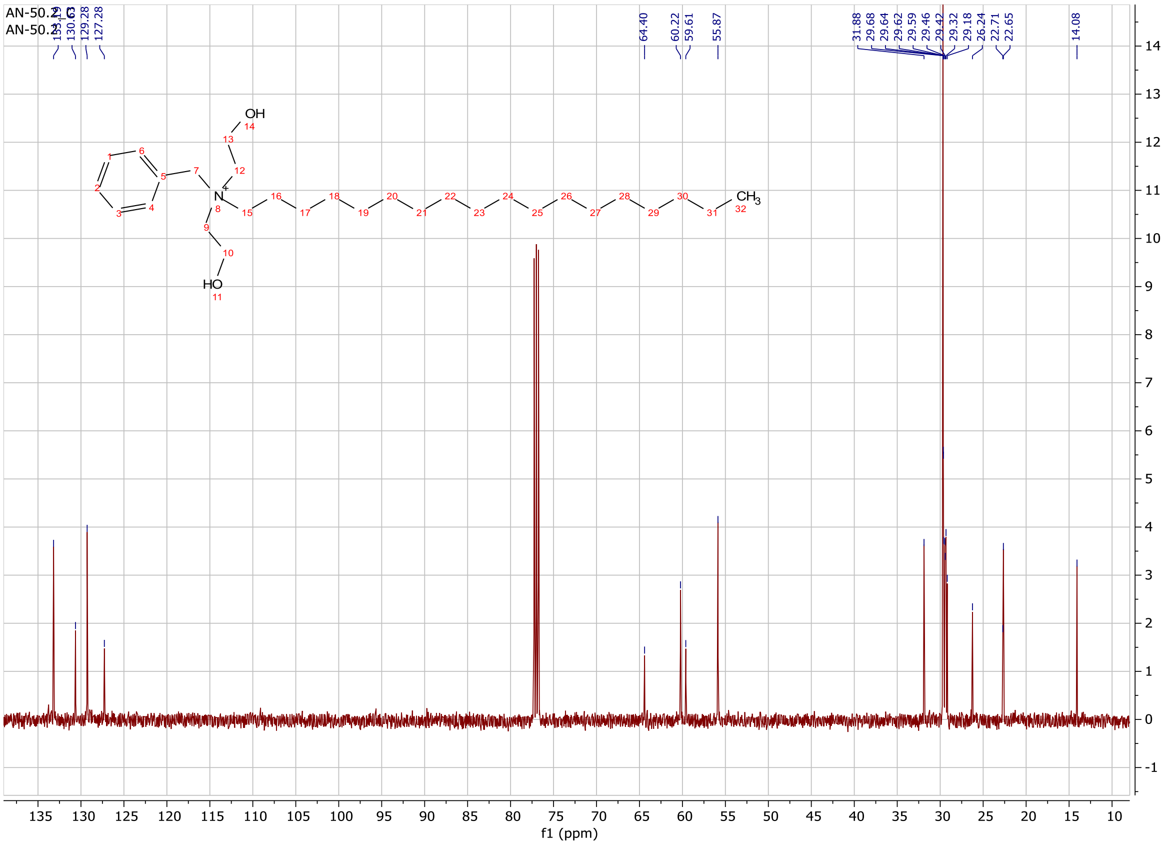

Figure S10. ^{13}C NMR (126 MHz, Chloroform-*d*) – **5e**

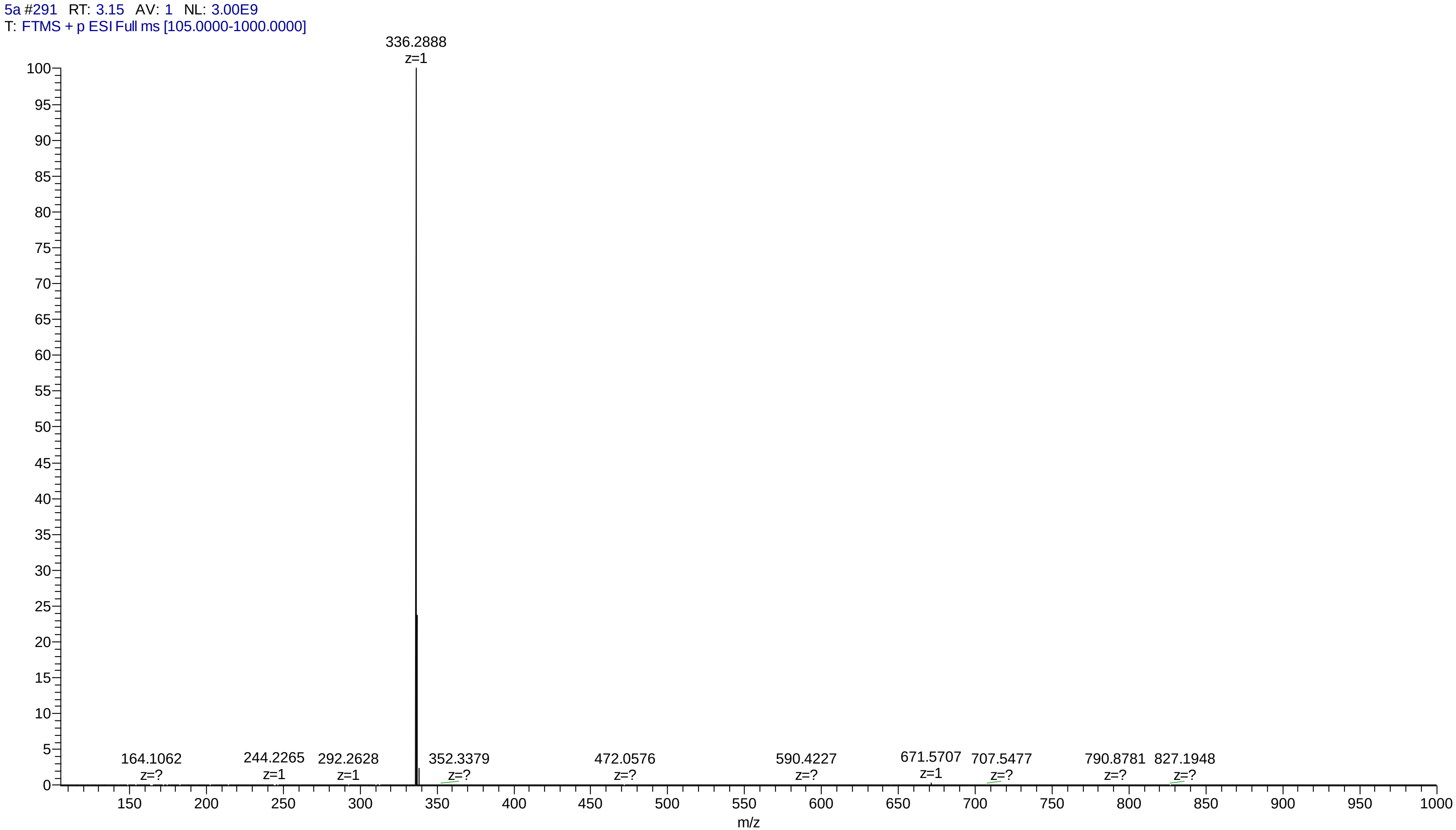

Figure S11. MS spectrum of **5a** in R_t 3.15 min

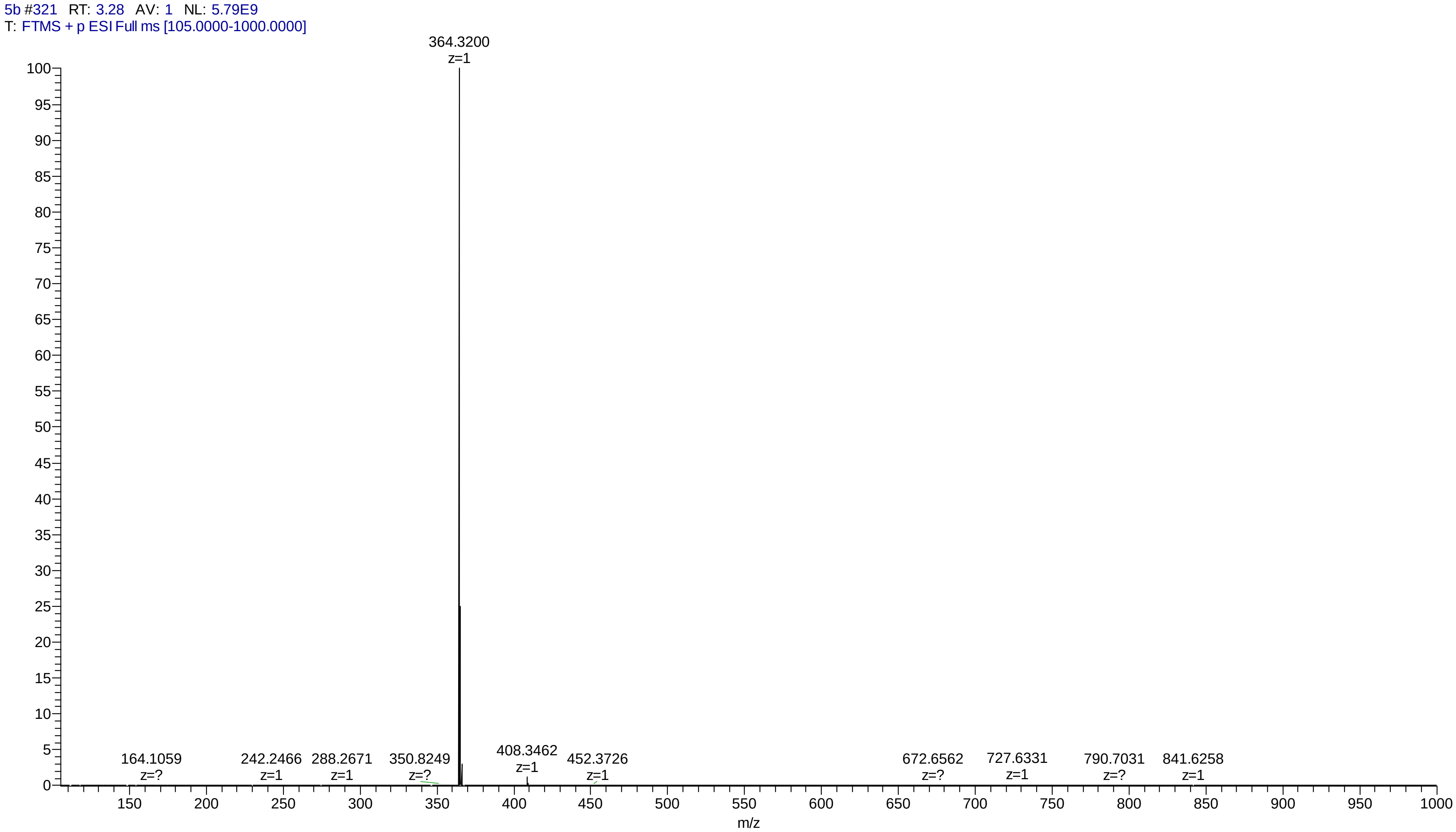

Figure S12. MS spectrum of **5b** in R_t 3.28 min

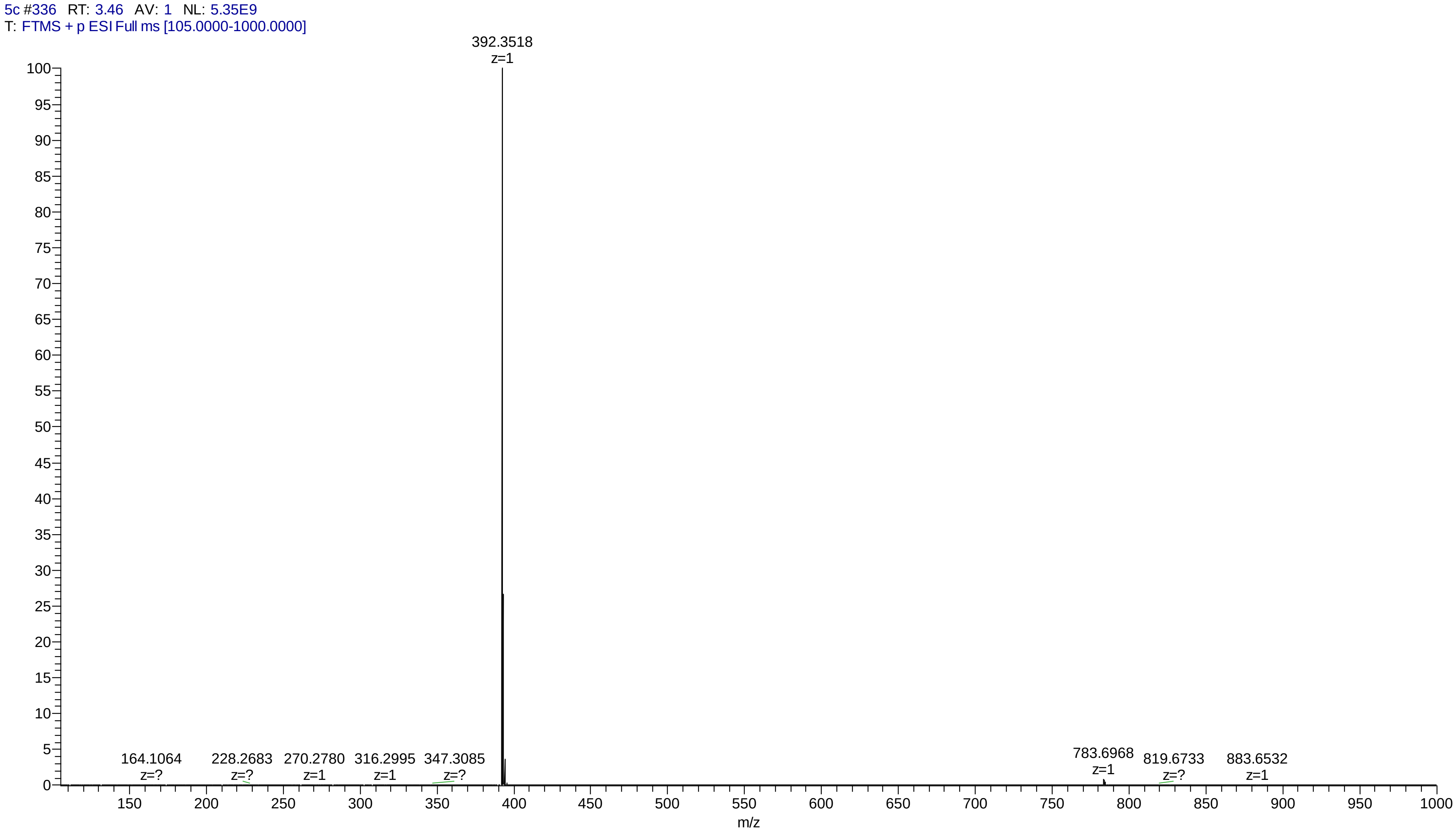

Figure S13. MS spectrum of **5c** in R_t 3.46 min

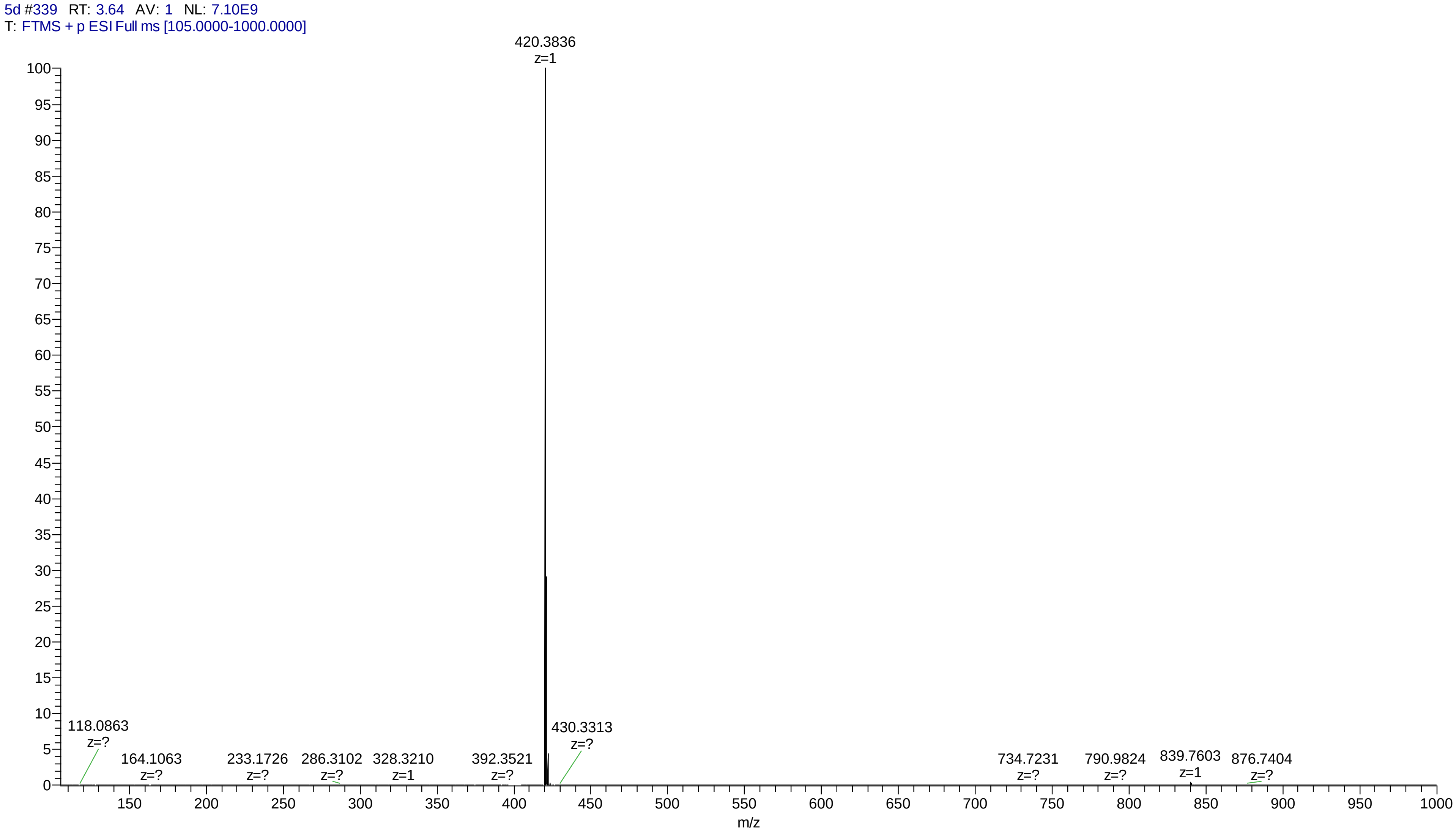

Figure S14. MS spectrum of **5d** in R_t 3.64 min

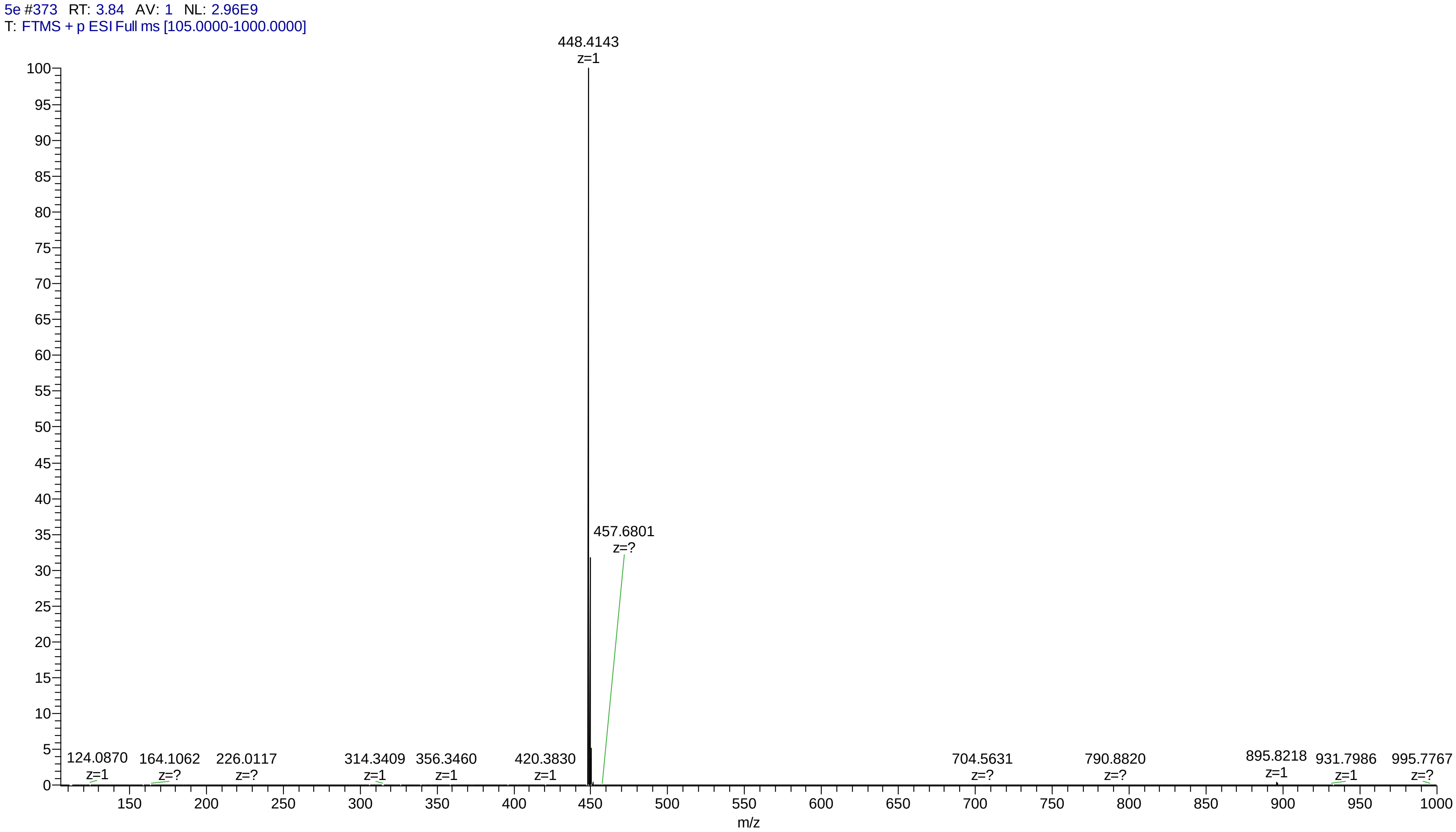

Figure S15. MS spectrum of **5e** in R_t 3.84 min
